# Cyclin Y regulates spatial learning and memory flexibility through distinct control of the actin pathway

**DOI:** 10.1038/s41380-022-01877-0

**Published:** 2022-11-25

**Authors:** Jiyeon Seo, Hongik Hwang, Heesung Sohn, Eunsil Cho, Sunmin Jung, Soohyun Kim, Seung Min Um, Ji Yeon Kim, Muwon Kang, Yuri Choi, Jong-Hwan Kim, Mirang Kim, Seon-Young Kim, Sun-Kyung Lee, Joohong Ahnn, Hyewhon Rhim, Dong-Gyu Jo, Eunjoon Kim, Mikyoung Park

**Affiliations:** 1 Brain Science Institute, Korea Institute of Science and Technology, Seoul, 02792 South Korea; 2 Center for Synaptic Brain Dysfunctions, Institute for Basic Science (IBS), Daejeon, 34141 South Korea; 3 Department of Biological Sciences, Korea Advanced Institute of Science and Technology (KAIST), Daejeon, 34141 South Korea; 4 Personalized Genomic Medicine Research Center, Korea Research Institute of Bioscience and Biotechnology, Daejeon, 34141 South Korea; 5 Department of Functional Genomics, Korea University of Science and Technology, Daejeon, 34141 South Korea; 6 Department of Life Sciences, Research Institute for Natural Sciences, School of Natural Science, Hanyang University, Seoul, 04763 South Korea; 7 Division of Bio-Medical Science and Technology, KIST School, Korea University of Science and Technology, Seoul, 02792 South Korea; 8 School of Pharmacy, Sungkyunkwan University, Suwon, 16419 South Korea

**Keywords:** Neuroscience, Molecular biology, Physiology

## Abstract

Spatial learning and memory flexibility are known to require long-term potentiation (LTP) and long-term depression (LTD), respectively, on a cellular basis. We previously showed that cyclin Y (CCNY), a synapse-remodeling cyclin, is a novel actin-binding protein and an inhibitory regulator of functional and structural LTP in vitro. In this study, we report that *Ccny* knockout (KO) mice exhibit enhanced LTP and weak LTD at Schaffer collateral-CA1 synapses in the hippocampus. In accordance with enhanced LTP, *Ccny* KO mice showed improved spatial learning and memory. However, although previous studies reported that normal LTD is necessary for memory flexibility, *Ccny* KO mice intriguingly showed improved memory flexibility, suggesting that weak LTD could exert memory flexibility when combined with enhanced LTP. At the molecular level, CCNY modulated spatial learning and memory flexibility by distinctively affecting the cofilin-actin signaling pathway in the hippocampus. Specifically, CCNY inhibited cofilin activation by original learning, but reversed such inhibition by reversal learning. Furthermore, viral-mediated overexpression of a phosphomimetic cofilin-S3E in hippocampal CA1 regions enhanced LTP, weakened LTD, and improved spatial learning and memory flexibility, thus mirroring the phenotype of *Ccny* KO mice. In contrast, the overexpression of a non-phosphorylatable cofilin-S3A in hippocampal CA1 regions of *Ccny* KO mice reversed the synaptic plasticity, spatial learning, and memory flexibility phenotypes observed in *Ccny* KO mice. Altogether, our findings demonstrate that LTP and LTD cooperatively regulate memory flexibility. Moreover, CCNY suppresses LTP while facilitating LTD in the hippocampus and negatively regulates spatial learning and memory flexibility through the control of cofilin-actin signaling, proposing CCNY as a learning regulator modulating both memorizing and forgetting processes.

## Introduction

Spatial learning is the process of recording the information required to navigate a location, and memory flexibility (also often called behavioral or cognitive flexibility) is the process of learning a new location that involves two courses, forming new memory and suppressing the previous memory. Synaptic plasticity, including long-term potentiation (LTP) and long-term depression (LTD), is generally considered as the cellular basis of learning and memory in the brain. Specifically, a myriad of preceding studies have reported the functional significance of LTP in mediating learning and memory with extensive molecular mechanisms [1, 2]. In addition, many studies have demonstrated that LTD is required for memory flexibility [3–6] and is also facilitated by learning per se [7–12]. Memory consolidation must be regulated within the physiological range through fine controls between memorizing and forgetting processes. Excessive memory strengthening and/or impaired forgetting processes likely contribute to a deficit in memory flexibility [13–15], which may lead to establishment of pathological conditions, such as post-traumatic stress disorder, autism spectrum disorder, schizophrenia, and Alzheimer’s disease [16–19]. Therefore, the maintenance of LTP, LTD, and memory strength within adequate physiological ranges is essential for the proper functioning of the brain in various cognitive and behavioral contexts.

Cyclin Y (CCNY), a cyclin family protein known to regulate the cell cycle in dividing cells [20–22], can regulate synaptic functions in terminally differentiated neuronal cells [23–26]. Specifically, CCNY is particularly enriched in the postsynaptic compartment in rat brains and inhibits the trafficking of α-amino-3-hydroxy-5-methyl-4-isoxazolepropionate (AMPA) receptor (AMPAR) to the synapse during glycine-induced LTP in cultured neurons [25]. In addition, the overexpression of CCNY, biolistically transfected in organotypic hippocampal slices, inhibits LTP at the Schaffer collateral-*Cornu Ammonis* 1 (CA1) synapses [25]. Furthermore, CCNY binds actin with high affinity and negatively regulates structural LTP by controlling actin dynamics [26]. Upon observation that CCNY regulates functional and structural LTP in cultured hippocampal neurons and organotypic slice systems [25, 26], we hypothesized that CCNY also mediates hippocampus-dependent cognitive function in vivo, including learning and memory, by regulating synaptic plasticity.

A strong correlation between LTD and memory flexibility has been demonstrated by showing that mice with impaired LTD but with normal LTP and/or spatial learning exhibit impaired memory flexibility [3, 4, 6, 27]. Furthermore, LTD was suggested to play a role in the extinction of prior memory when new learning occurs during the process of memory flexibility [28]. However, it is still unclear whether intact LTD is a particular requirement for memory flexibility, and whether LTP and LTD distinctively play a role in the memorizing and forgetting processes.

In the present study, *Ccny* KO mice exhibited enhanced LTP and reduced LTD at Schaffer collateral-CA1 synapses in the hippocampus, indicating a shifted spectrum in synaptic plasticity toward potentiation, and showed improved spatial learning in the Morris water maze (MWM) task as well as improved memory flexibility in the reversal learning phase of the MWM task and in a delayed nonmatch to place (DNMTP) T-maze task. Notably, transcriptome and immunoblot analyses revealed that LIM domain kinase 1 (LIMK1)-cofilin-actin signaling is differentially regulated in response to original learning and reversal learning in *Ccny* KO mice. Mice overexpressing a phosphomimetic cofilin-S3E in the hippocampal CA1 region, recapitulating the basal state of *Ccny* KO mice, also showed enhanced LTP and reduced LTD at Schaffer collateral-CA1 synapses as well as improved spatial learning and memory flexibility. *Ccny* KO mice overexpressing a non-phosphorylatable cofilin-S3A in the hippocampal CA1 regions showed reversion of the synaptic plasticity, spatial learning, and memory flexibility phenotypes observed in *Ccny* KO mice. Taken together, our findings demonstrate that reduced LTD can mediate normal and even enhanced memory flexibility when combined with normal and enhanced LTP, respectively, suggesting a crosstalk between LTP and LTD in the context of memory flexibility. In addition, this study suggests that CCNY is a learning regulator that modulates both memorizing and forgetting processes, and that spatial learning and memory flexibility are differentially modulated through the CCNY-mediated actin signaling pathway at the organismal level.

## Results

### Neuronal excitability and basal excitatory synaptic properties remain unaltered in *Ccny* KO mice

**Fig. 1:**
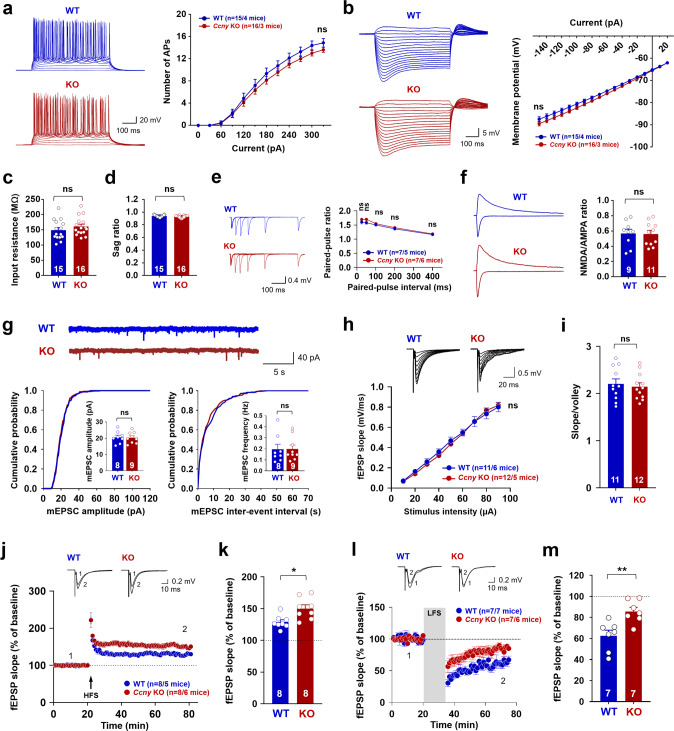
*Ccny* knockout (KO) mice exhibit enhanced long-term potentiation (LTP) and moderate long-term depression (LTD). **a**−**i** Electrophysiological characteristics of *Ccny* KO mice. **a** Neuronal intrinsic excitability is normal in *Ccny* KO mice. Data represent mean ± SEM of the number of action potentials triggered at each current step. The frequency of action potential firing was measured upon injecting a current step ranging from 0 to 330 pA in increments of 30 pA. ns, not significant compared to wild-type (WT) at each current step, Student’s unpaired *t* test. n = 15 and 16 cells from 4 WT and 3 KO mice, respectively. Representative action potentials recorded from WT and *Ccny* KO mice are shown. Scale bars, 20 mV, 100 ms. **b**–**d** The current-voltage relationship is not altered in *Ccny* KO mice. **b** Data represent mean ± SEM of the peak membrane potential measured at each current step. Membrane potential was measured upon injecting a current step ranging from 20 to −150 pA. ns, not significant compared to WT at each current step, Student’s unpaired *t* test. *n* = 15 and 16 cells from 4 WT and 3 KO mice, respectively. Representative voltage responses recorded from WT and *Ccny* KO mice are shown. Scale bars, 5 mV, 100 ms. Input resistance (**c**) and sag ratio (**d**) were calculated from the current-voltage relationship. Data represent mean ± SEM. ns, not significant, Student’s unpaired *t* test. **e** Paired-pulse ratio (PPR) remains unchanged in *Ccny* KO mice. Data represent mean ± SEM of PPR at each time interval. Two consecutive field excitatory postsynaptic potentials (fEPSPs) were evoked at Schaffer collateral-CA1 synapses with various time intervals (25, 50, 100, 200, and 400 ms). PPR was calculated by dividing the peak of the second fEPSP by that of the first one. ns, not significant, Student’s unpaired *t* test at each time interval. Representative fEPSP traces recorded from WT and *Ccny* KO mice are shown. Scale bars, 0.4 mV, 100 ms. **f** NMDA/AMPA ratio at hippocampal Schaffer collateral-CA1 synapses is normal in *Ccny* KO mice. Data represent mean ± SEM of NMDA/AMPA ratio. AMPAR-mediated currents recorded at −70 mV were measured at the peak amplitude, and NMDAR-mediated currents recorded at +40 mV were measured at 60 ms after stimulation. ns, not significant, Student’s unpaired *t* test. *n* = 9 and 11 cells from 4 WT and 3 KO mice, respectively. Representative excitatory postsynaptic currents (EPSCs) recorded at −70 mV (downward current) and +40 mV (upward current) from WT and *Ccny* KO mice. Scale bars, 100 pA, 100 ms. **g** Miniature EPSCs (mEPSCs) are not altered in hippocampal CA1 neurons from *Ccny* KO mice. (Upper) Representative recordings of AMPAR mEPSCs in hippocampal CA1 neurons from WT and *Ccny* KO mice. Scale bars, 40 pA, 5 s. (Lower) Both mEPSC amplitude (left) and frequency (right) are not altered by chronic *Ccny* KO. Cumulative distribution function plots of AMPAR mEPSC amplitude (left) and inter-event intervals (right). Insets, Data represent mean ± SEM. ns, not significant, Student’s unpaired *t* test. *n* = 8 and 9 cells from 5 animals each for WT and KO mice, respectively. **h**, **i** The input-output relationship is not altered at Schaffer collateral-CA1 synapses in *Ccny* KO mice. **h** fEPSPs were measured by stimulating the Schaffer collateral afferents with various stimulus intensity. (Upper) Representative fEPSP traces recorded from WT and *Ccny* KO mice with a series of increasing input stimulus ranging from 10 to 90 μA. Scale bars, 0.5 mV, 20 ms. (Lower) Data represent mean ± SEM of fEPSP slope at each stimulus intensity. ns, not significant, *P* = 0.669 (10 μA), *P* = 0.289 (20 μA), *P* = 0.430 (30 μA), *P* = 0.255 (40 μA), *P* = 0.403 (50 μA), *P* = 0.772 (60 μA), *P* = 0.935 (70 μA), *P* = 0.540 (80 μA), and *P* = 0.765 (90 μA) compared to WT at each stimulus intensity, Student’s unpaired *t* test. *n* = 11 and 12 slices from 6 WT and 5 KO mice, respectively. **i** Data represent mean ± SEM of the ratio of slope to volley. The ratio of fEPSP slope to the fiber volley amplitude at the stimulus strength that evokes 40% of the maximal fEPSP amplitude. ns, not significant, *P* = 0.662, Student’s unpaired *t* test. *n* = 11 and 12 slices from 6 WT and 5 KO mice, respectively. **j**, **k** LTP at Schaffer collateral-CA1 synapses is enhanced in *Ccny* KO mice. **j** High-frequency stimulation (HFS)-induced LTP is enhanced in *Ccny* KO mice. The magnitude of LTP was quantified as an increase in the fEPSP slope relative to the baseline. Representative fEPSP traces from WT and KO mice before (1) and after (2) LTP induction are shown. Scale bars, 0.2 mV and 10 ms. *n* = 8 and 8 slices from 5 WT and 6 KO mice, respectively. Data represent mean ± SEM of fEPSP slope. **k** Data represent mean ± SEM of averages of fEPSP slopes during the last 10 min (71−80 min) of the recordings in (**j**). *P < 0.05 as indicated, Student’s unpaired *t* test. **l**, **m** LTD at Schaffer collateral-CA1 synapses is reduced but still expressed in *Ccny* KO mice. **l** *Ccny* KO mice exhibit reduced hippocampal LTD induced by low-frequency stimulation (1 Hz, 900 pulses) at Schaffer collateral-CA1 synapses. The magnitude of LTD was quantified as a decrease in the fEPSP slope relative to the baseline. Data represent mean ± SEM. *n* = 7 and 7 slices from 7 WT and 6 KO mice, respectively. Representative fEPSP traces from WT and KO mice before (1) and after (2) LTD induction. Scale bars, 0.2 mV and 10 ms. **m** Data represent mean ± SEM of averages of fEPSP slopes during the last 10 min (66-75 min) of the recordings in (**l**). **P < 0.005 as indicated, Student’s unpaired *t* test. See also Supplementary Fig. S2.

We first investigated the role of CCNY in hippocampal synaptic plasticity and cognitive functions by characterizing electrophysiological features in *Ccny* KO mice [29]. The frequency of action potential firing triggered by current injection in CA1 neurons was not altered in *Ccny* KO mice (Fig. 1a). The current-voltage relationship (Fig. 1b), input resistance (Fig. 1c), and sag ratio (Fig. 1d) remained unaffected, demonstrating that intrinsic neuronal excitability is normal in *Ccny* KO mice. Moreover, the paired-pulse ratio (PPR) was not significantly altered in *Ccny* KO mice (Fig. 1e), indicating that presynaptic release probability at Schaffer collateral-CA1 synapses in *Ccny* KO mice remains comparable to that of wild-type (WT) mice. This result is in agreement with the finding that CCNY expression is absent in the synaptic vesicle-enriched (LP2) presynaptic fraction, where a presynaptic protein synaptophysin exists in the mouse (Supplementary Fig. S1a) and rat forebrain [25]. CCNY was enriched in postsynaptic fractions of the mouse forebrain, including the synaptic plasma membrane (SPM) and the postsynaptic density (PSD) fractions, where postsynaptic proteins, such as GluA1, a subunit of AMPARs, and PSD-95, are located (Supplementary Fig. S1a).

We next examined the function of AMPAR and *N-methyl*-D-aspartate (NMDA) receptor (NMDAR). The ratio of NMDAR- to AMPAR-mediated synaptic transmission (NMDA/AMPA ratio) was normal in *Ccny* KO mice (Fig. 1f), and the amplitude and frequency of miniature excitatory postsynaptic currents (mEPSCs) at Schaffer collateral-CA1 synapses were not altered (Fig. 1g). In addition, the input-output relationship between presynaptic stimulus intensity and the slope of the resulting field excitatory postsynaptic potentials (fEPSPs) against the presynaptic stimulus intensity (Fig. 1h) and the ratio of fEPSP slope to the fiber volley amplitude (Fig. 1i) were also not affected in *Ccny* KO mice. These results together indicate that basal excitatory synaptic transmission remained unchanged in *Ccny* KO mice. In support of these findings, the postsynaptic subcellular localizations of GluA1 and PSD-95 were not altered in the forebrain of *Ccny* KO mice (Supplementary Fig. S1b, c). The unaltered basal excitatory synaptic transmission in *Ccny* KO mice is rather contradictory to that reported in a previous study showing that a short hairpin RNA (shRNA)-mediated knockdown of CCNY increases the amplitude but not the frequency of mEPSCs in cultured rat hippocampal neurons [26]. This difference could be due to the use of different systems; the chronic depletion of CCNY in *Ccny* KO mice might be systemically complemented in vivo, whereas 3- to 4-day knockdown of CCNY in cultured neuronal system rather exhibits an acute response to reduced CCNY levels. Together, these results suggest that the complete depletion of CCNY does not affect intrinsic neuronal excitability and basal excitatory synaptic transmission in the hippocampus.

### CCNY regulates hippocampal LTP and LTD at Schaffer collateral-CA1 synapses

Hippocampal synaptic plasticity, including LTP and LTD, is widely considered as a fundamental mechanism underlying cognitive brain functions of learning and memory [5, 9, 10, 30–33]. We found that the magnitude of LTP induced by high-frequency stimulation (HFS) at Schaffer collateral-CA1 synapses was slightly but significantly enhanced in acute hippocampal slices prepared from *Ccny* KO mice compared to WT littermates (Fig. 1j, k). Furthermore, LTD induced by low-frequency stimulation (900 pulses, 1 Hz, 15 min) at Schaffer collateral-CA1 synapses was expressed with reduced magnitude in *Ccny* KO mice compared to WT littermates (Fig. 1l, m). In contrast, both voltage-gated calcium channel (VGCC)-dependent LTP (Supplementary Fig. S2a, b) and metabotropic glutamate receptor (mGluR)-dependent LTD (Supplementary Fig. S2c, d) were normal in *Ccny* KO mice. Collectively, these results indicate that *Ccny* KO mice possess a shifted spectrum of synaptic plasticity toward potentiation in the hippocampus and specifically affect the NMDAR-dependent form of synaptic plasticity, thereby potentially affecting hippocampus-dependent cognitive functions, such as spatial learning and memory flexibility.

### Hippocampus-dependent spatial learning is enhanced in *Ccny* KO mice

**Fig. 2:**
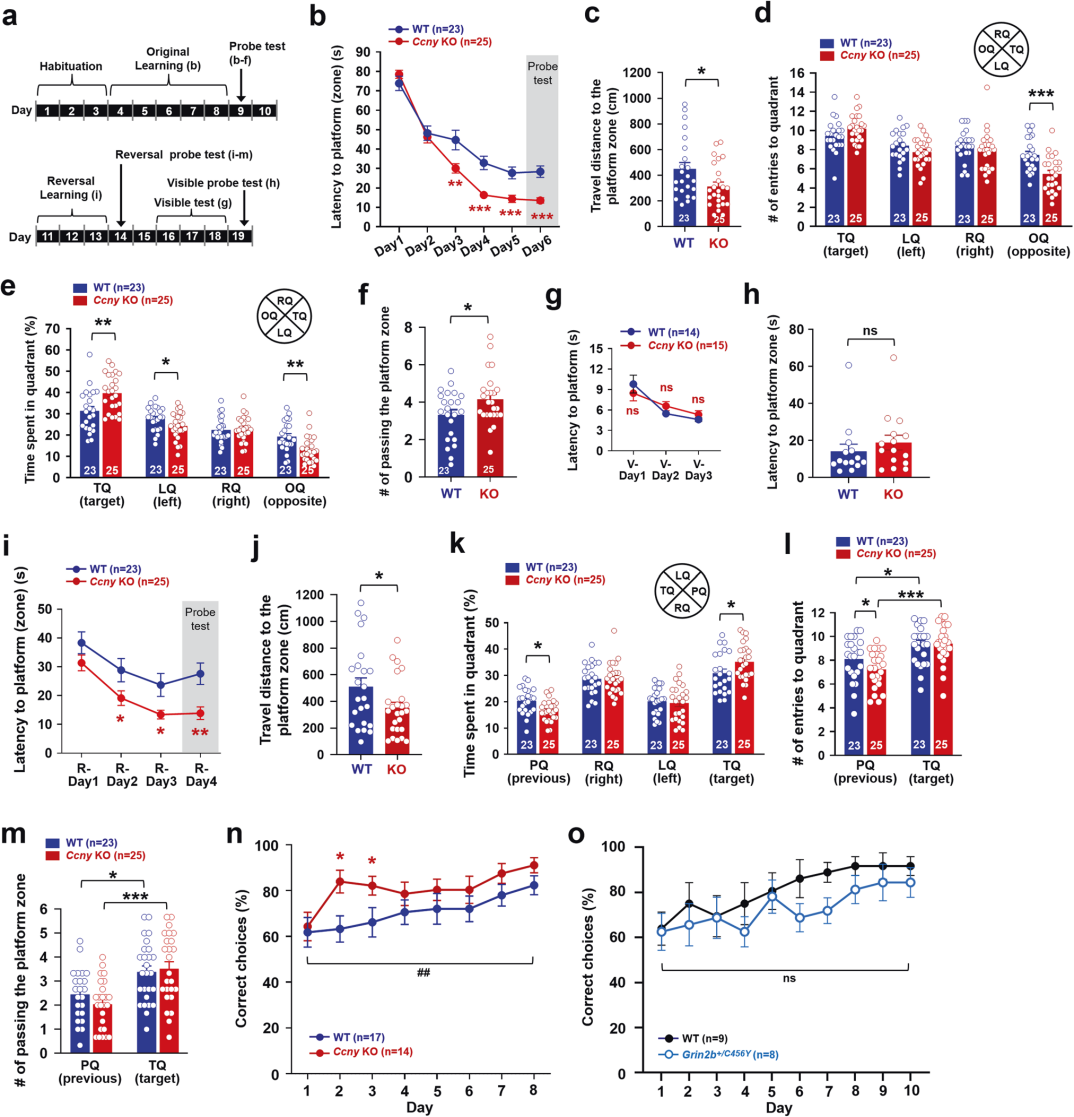
Spatial learning and memory flexibility are improved in *Ccny* KO mice. **a** Schematic diagram for the Morris water maze (MWM) task, including original (Fig. 2b−f) and reversal (Fig. 2i−m) learning and visible platform (Fig. 2g, h) tests. **b** Learning curve of latency to platform during the original training sessions, Day 1 to 5, and latency to platform zone during the probe test session on Day 6 performed in the MWM after platform removal (gray shaded area). Data represent mean ± SEM. **P < 0.005, ***P < 0.0005 as indicated, Student’s unpaired *t* test. Data obtained from three cohorts. **c** Travel distance to platform zone during the probe test on Day 6 performed in the MWM after platform removal. *P < 0.05, Student’s unpaired *t* test. **d**, **e** The number of entries to (**d**) and time spent in (**e**) each quadrant during the probe test on Day 6 performed in the MWM after platform removal. TQ, target quadrant; LQ, quadrant left to the TQ; RQ, quadrant right to the TQ; OQ, quadrant opposite to the TQ. *P < 0.05, **P < 0.005, ***P < 0.0005 as indicated, Student’s unpaired *t* test. **f** The number of passing the platform zone during the probe test on Day 6 performed in the MWM after platform removal. *P < 0.05, Student’s unpaired *t* test. **g**, **h** Latency to platform during the visible sessions, V-Day 1 to 3 (**g**) and latency to platform zone during the visible probe test on V-Day 4 (**h**). Data represent mean ± SEM. ns, not significant, *P* = 0.223 (V-Day 1), *P* = 0.100 (V-Day 2), and *P* = 0.143 (V-Day 3) in (**g**), *P* = 0.203 in (**h**), Student’s unpaired *t* test. See also Supplementary Fig. S3. **i**−**m** *Ccny* KO mice exhibit an improved memory flexibility in reversal learning of the MWM task. **i** Latency to platform during the reversal learning training sessions, R-Day 1 to 3, and latency to platform zone during the reversal learning probe test on R-Day 4 (gray shaded area). Data represent mean ± SEM. *P < 0.05, **P < 0.005 as indicated, Student’s unpaired *t* test. Data obtained from three cohorts. **j** Travel distance to the platform zone during the reversal learning probe test on R-Day 4 performed in the MWM after platform removal. *P < 0.05, Student’s unpaired *t* test. **k** Time spent in each quadrant during the reversal learning probe test on R-Day 4 performed in the MWM after platform removal. Data represent mean ± SEM. PQ, previous target quadrant; TQ, target quadrant; LQ, quadrant left to the TQ; RQ, quadrant right to the TQ. *P < 0.05 as indicated, Student’s unpaired *t* test. **l** The number of entries to the previous target (PQ) and current target quadrant (TQ) during the reversal learning probe test on R-Day 4 performed in the MWM after platform removal. Data represent mean ± SEM. *P < 0.05, ***P < 0.0005 as indicated, Student’s unpaired *t* test. **m** The number of passing the platform zone during the reversal learning probe test on R-Day 4 performed in the MWM after platform removal. Data represent mean ± SEM. *P < 0.05, ***P < 0.0005 as indicated, Student’s unpaired *t* test. **n** *Ccny* KO mice exhibit improved memory flexibility in a delayed nonmatch to place (DNMTP) T-maze task. Data represent mean ± SEM of percentage of correct choices. *P < 0.05, Student’s unpaired *t* test. ^##^*P* < 0.005 as indicated, repeated-measures two-way ANOVA, the effect of genotype, F (1, 29) = 9.874. See also Supplementary Fig. S4. **o** Memory flexibility in GluN2B-C456Y-mutant mice is comparable to the littermate WT in a DNMTP T-maze task. Data represent mean ± SEM of percentage of correct choices. Student’s unpaired *t* test. ns, not significant, repeated-measures two-way ANOVA, the effect of genotype, F (1, 15) = 2.244. See also Supplementary Fig. S4.

We next investigated whether CCNY regulates hippocampus-dependent spatial learning and memory using the MWM task equipped with surrounding spatial cues (Fig. 2a). The latency to platform was significantly shorter on days 3, 4, and 5 in *Ccny* KO mice compared to WT littermates during the five consecutive training days (Fig. 2b), indicating an enhanced learning ability in *Ccny* KO mice. In addition, during the probe test on day 6, the latency (Fig. 2b) and travel distance (Fig. 2c) to the platform zone where the platform was originally located was significantly shorter in *Ccny* KO mice. Even though the number of entries to the target quadrant was not significantly different between WT and *Ccny* KO mice (Fig. 2d), the time spent in the target quadrant was increased in *Ccny* KO mice compared to WT littermates (Fig. 2e). Furthermore, the frequency of passing the platform zone was increased in *Ccny* KO mice compared to WT littermates (Fig. 2f). These results together indicate an enhancement of spatial memory ability in *Ccny* KO mice. In the visible platform test, which is a hippocampus-independent non-spatial learning task, the performance of *Ccny* KO mice was comparable to that of WT mice in terms of the latency to platform during the training sessions (Fig. 2g) and to the platform zone during the probe test (Fig. 2h), suggesting that the improved spatial learning and memory of *Ccny* KO mice in the MWM task (Fig. 2b−f) were not due to the enhanced motivation for escape or improved vision and/or motor skills.

In addition, the total distance moved, the time spent in the center, and the number of center entries in an open field task were not different between WT and *Ccny* KO mice (Supplementary Fig. S3a−h), further supporting that basal locomotion, anxiety, and willingness to explore were not affected in *Ccny* KO mice compared to WT littermates. Moreover, no significant difference was observed between WT and *Ccny* KO mice in a novel object recognition task (Supplementary Fig. S3i−k) used to test non-spatial memory [34, 35] or in a passive avoidance task (Supplementary Fig. S3l, m) used to assess aversive associative memory [36]. These results together demonstrate that hippocampus-dependent spatial learning and memory is specifically improved in *Ccny* KO mice and suggest future studies exploring the roles of CCNY in other spectrums of learning behaviors and/or other behavioral/non-behavioral functions in other brain regions.

### Memory flexibility is improved in *Ccny* KO mice and normal in *Grin2b*^+/C456Y^ mice

LTD is associated with behavioral and memory flexibility [3–6, 9, 10, 27, 28]. Because hippocampal LTD was significantly altered in *Ccny* KO mice (Fig. 1l, m), we further investigated whether CCNY is involved in the regulation of memory flexibility. To this end, a spatial reversal learning task was conducted by moving the hidden platform to a new location (i.e., the quadrant opposite to the previously memorized quadrant) following the original learning task in the MWM. The latency to platform was significantly shorter on days 2 and 3 during the training sessions in *Ccny* KO mice (Fig. 2i). In addition, on day 4 of the reversal learning probe test, the latency (Fig. 2i) and travel distance (Fig. 2j) to the platform zone were also significantly reduced in *Ccny* KO mice, and the time spent in the target quadrant was significantly increased in *Ccny* KO mice compared to WT littermates (Fig. 2k). Furthermore, both the time spent in the previous target quadrant (Fig. 2k) and the frequency of entries to the previous target quadrant during the reversal learning probe test (Fig. 2l) were significantly decreased in *Ccny* KO mice compared to WT littermates. Both WT littermates and *Ccny* KO mice more frequently entered to the target quadrant (Fig. 2l) and target platform zone (Fig. 2m) than to the previous quadrant (Fig. 2l) and previous platform zone (Fig. 2m), respectively, but the degrees of increase in the frequency of entries to the target quadrant and target platform zone were much higher in *Ccny* KO mice than in WT littermates (Fig. 2l, m). Altogether, these results indicate that memory flexibility (or new learning and memory ability) is improved in *Ccny* KO mice.

We further investigated whether the lack of CCNY also influences the performance in the DNMTP T-maze task as an independent test to examine hippocampus-dependent memory flexibility [3, 27, 28, 37]. Similar to the reversal learning of MWM task which requires memory flexibility to learn and memorize new spatial information, the location of rewards for the choice runs in the DNMTP T-maze task alternates between the right and left arms in each trial, thus requiring memory flexibility to make a correct choice. Consistent with the enhanced reversal learning (Fig. 2i−m), *Ccny* KO mice also showed improved learning flexibility in the DNMTP T-maze task (Fig. 2n). In agreement with this finding, both travel distance (Supplementary Fig. S4a) and travel time (Supplementary Fig. S4b) taken to make a correct choice in the DNMTP T-maze task were shorter in *Ccny* KO mice compared to WT littermate. The body weights of the mice subjected to this task were maintained at approximately 80% of the initial body weights during the handling period throughout the experiments (Supplementary Fig. S4c). Because the animal behaviors in the open field task were not significantly different between WT and *Ccny* KO mice (Supplementary Fig. S4a−h), the improved memory flexibility in *Ccny* KO mice was not due to a change in basal locomotion, anxiety, or willingness to explore. Collectively, our findings indicate that even reduced LTD in *Ccny* KO mice (Fig. 1l, m) supports memory flexibility of both reversal learning in MWM (Fig. 2i−m) and the DNMTP T-maze task (Fig. 2n).

To further examine the notion that reduced LTD is sufficient to support memory flexibility, we performed a DNMTP T-maze task using GluN2B-C456Y-mutant mice (Grin2b^+/C456Y^) that exhibited reduced LTD induced by low-frequency stimulation but normal LTP at Schaffer collateral-CA1 synapses (i.e., presenting an altered spectrum in synaptic plasticity) along with normal original and reversal learning in MWM [38]. Despite the reduced hippocampal LTD [38], the GluN2B-C456Y-mutant mice showed memory flexibility comparable to WT littermates in the DNMTP T-maze task (Fig. 2o), consistent with the normal reversal learning of MWM observed in GluN2B-C456Y-mutant mice [38], confirming that reduced LTD is sufficient to support memory flexibility. In agreement with this finding, both travel distance (Supplementary Fig. S4d) and travel time (Supplementary Fig. S4e) taken to make the correct choice in GluN2B-C456Y-mutant mice were comparable to WT littermates. The body weights of the mice were maintained at approximately 80% of their initial body weight during the handling period throughout the experiments (Supplementary Fig. S4f). Considering that the GluN2B-C456Y-mutant mice showed a difference in terms of normal LTP and normal original learning when compared to *Ccny* KO mice, the improved memory flexibility in *Ccny* KO mice seems to be accounted for by the enhanced LTP and improved original learning ability.

**Fig. 3:**
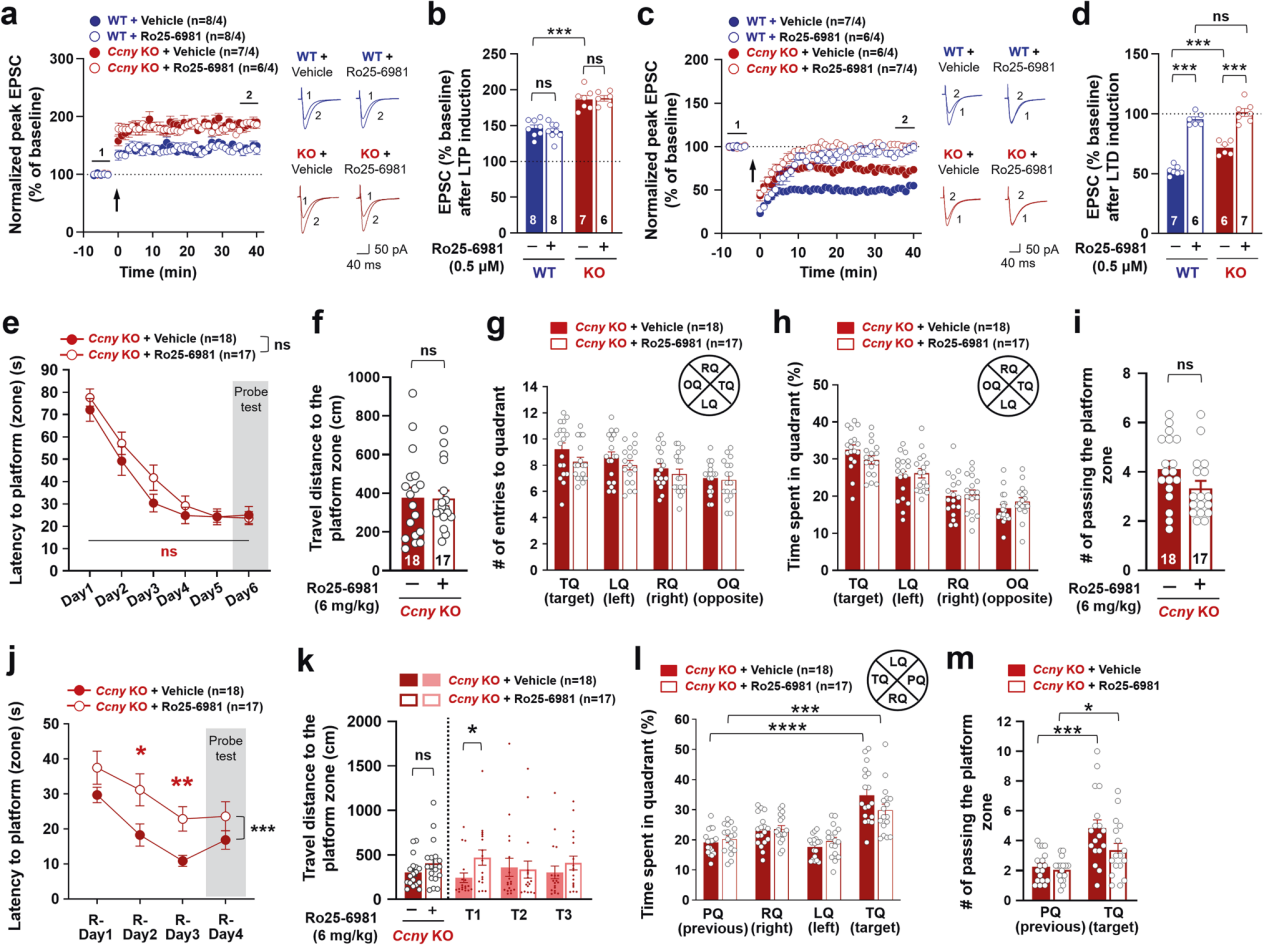
The specific LTD inhibitor Ro 25-6981 inhibits LTD and memory flexibility but does not affect LTP and spatial learning in *Ccny* KO mice. **a**, **b** Ro25-6981 (0.5 μM) treatment does not affect the pairing-induced LTP in both WT and *Ccny* KO mice. **a** Data represent mean ± SEM of the peak EPSC amplitude relative to the baseline. LTP was induced by pairing stimulation (2 Hz for 90 s with 0 mV holding) at Schaffer collateral-CA1 synapses. *n* = 8, 8, 7, and 6 slices from 4 mice in each of 4 experimental groups. Representative evoked EPSC traces before (1) and after (2) LTP induction are shown. Scale bars, 50 pA and 40 ms. **b** Data represent mean ± SEM of evoked EPSC amplitudes during the last 5 min (35−40 min) of the recordings in (**a**). ns, not significant, ***P < 0.0005 as indicated, one-way ANOVA followed by Tukey’s multiple comparisons test. **c**, **d** Ro25-6981 (0.5 μM) treatment inhibits the pairing-induced LTD in both WT and *Ccny* KO mice. (**c**) Data represent mean ± SEM of the peak EPSC amplitude relative to the baseline. LTD was induced by a pairing protocol (5 Hz for 3 min with −40 mV holding) at Schaffer collateral-CA1 synapses. *n* = 7, 6, 6, and 7 slices from 4 mice in each of 4 experimental groups. Representative evoked EPSC traces before (1) and after (2) LTD induction are shown. Scale bars, 50 pA and 40 ms. **d** Data represent mean ± SEM of the evoked EPSC amplitudes during the last 5 min (35−40 min) of the recordings in (**c**). ns, not significant, ***P < 0.0005 as indicated, one-way ANOVA followed by Tukey’s multiple comparisons test. **e**−**i** The intraperitoneal injection of Ro25-6981 (6 mg/kg) into *Ccny* KO mice did not affect original learning in the MWM task. **e** Learning curve of latency to platform (zone) during the MWM task. Data represent mean ± SEM. colored ns, not significant, Student’s unpaired *t* test at individual days. ns, not significant as indicated, two-way ANOVA. **f** Travel distance to platform zone during the probe test on Day 6. ns, not significant, Student’s unpaired *t* test. **g**, **h** The number of entries to (**g**) and time spent in (**h**) each quadrant during the probe test on Day 6. **i** The number of passing the platform zone during the probe test on Day 6. ns, not significant, Student’s unpaired *t* test. **j**−**m** Intraperi*t*oneal injection of Ro25-6981 (6 mg/kg) into *Ccny* KO mice inhibited memory flexibility in reversal learning of the MWM task. **j** Latency to the platform (zone) during the reversal learning. Data represent mean ± SEM. *P < 0.05, **P < 0.005 as indicated, Student’s unpaired *t* test. ***P < 0.0005 as indicated, two-way ANOVA. **k** Travel distance to the platform zone during the reversal learning probe test on R-Day 4. ns, not significant, *P < 0.05 as indicated, Student’s unpaired *t* test. Bars on the left side of the dotted line are the averaged data of T1 (Trial 1), T2 (Trial 2), and T3 (Trial 3). **l** Time spent in each quadrant during the reversal learning probe test on R-Day 4. Data represent mean ± SEM. ***P < 0.0005, ****P < 0.0000005 as indicated, Student’s unpaired *t* test. **m** The number of passing the platform zone during the reversal learning probe *t*est on R-Day 4. Data represent mean ± SEM. *P < 0.05, ***P < 0.0005 as indicated, Student’s unpaired *t* test.

To further support the requirement of residual LTD in *Ccny* KO mice for the improved memory flexibility, the residual LTD in *Ccny* KO mice was completely blocked using a selective GluN2B antagonist and a specific LTD inhibitor, Ro25-6981 [4, 39]. Ro25-6981 treatment did not affect LTP (Fig. 3a, b), whereas it completely removed residual LTD in both WT and *Ccny* KO mice (Fig. 3c, d). In agreement with LTP results, Ro25-6981 administration to *Ccny* KO mice did not affect spatial learning in the MWM task, as it resulted in no significant changes in the latency to the platform (zone) (Fig. 3e), travel distance (Fig. 3f), number of entries to the target quadrant (Fig. 3g), time spent in the target quadrant (Fig. 3h), and frequency of passing the platform zone (Fig. 3i). In agreement with the notion that the requirement of the residual LTD in *Ccny* KO mice is required for the improved memory flexibility, latency to the platform (zone) was significantly delayed (Fig. 3j) and travel distance to the platform zone tended to increase with Ro25-6981 treatment (Fig. 3k). Both Ro25-6981-treated and untreated *Ccny* KO mice spent more time in the target quadrant than in the previous quadrant (Fig. 3l) and more frequently passed the target platform zone than the previous target platform zone (Fig. 3m). However, the degrees to which both the time spent in the target quadrant and the frequency of passing to the target platform zone increased were significantly lower in Ro25-6981-treated *Ccny* KO mice than in vehicle-treated *Ccny* KO mice (Fig. 3l, m). These data indicate that the enhanced memory flexibility in *Ccny* KO mice is dependent on the residual LTD. Taken together, these findings suggest that to improve memory flexibility, it is critical to facilitate not only the extinction or weakening of the previously encoded spatial memory, but also the formation of new spatial memory. The facilitation of the weakening of previously acquired memory information and the formation of new spatial memory are presumably mediated by weakened LTD and enhanced LTP, respectively, in *Ccny* KO mice.

### Original learning upregulates genes related to the actin cytoskeleton, synaptic plasticity, or learning in WT mice with similar changes in untrained *Ccny* KO mice

Spatial learning and memory are correlated with spine morphology and plasticity [2, 40, 41], which requires actin remodeling [42–48]. CCNY is an actin-binding protein and regulates spine plasticity induced by LTP through the cofilin-actin signaling pathway [26], implying the functional significance of CCNY in modulating spatial learning and memory through the actin signaling pathway.

**Fig. 4:**
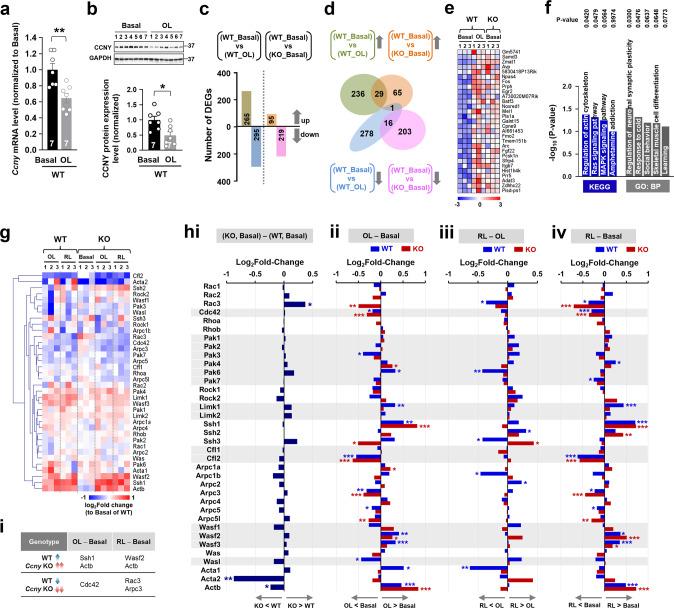
Molecular changes induced by spatial learning training in WT mice are similarly associated with those in untrained *Ccny* KO mice. **a**, **b** Transcript (**a**) and protein (**b**) levels of *Ccny* in the hippocampus are reduced after original learning in WT mice. Data represent mean ± SEM. *P < 0.05, **P < 0.005 as indicated, Student’s unpaired *t* test. *Ccny* mRNA levels (**a**) were measured by qu**a**ntitative real-time polymerase chain reaction (qRT-PCR), and CCNY protein expression levels (b) were quantified from the immunoblots (Upper in **b**). **c**, **d**) The number (**c**) and Venn diagram (**d**) of differentially expressed genes (DEGs) that are upregulated or downregulated compared to WT mice without training (WT_Basal), to *Ccny* KO mice without training (KO_Basal), or to WT mice after original learning (WT_OL). **e** Heatmap of 29 DEGs that are upregulated in both WT mice after original learning and KO mice without training, compared to WT mice without training. The values of log_2_CPM were normalized to the value ranges from a minimum of −3.0 to a maximum of +3.0. CPM, counts per million. **f** The pathway of “regulation of actin cytoskeleton” by the Kyoto Encyclopedia of Genes and Genomes enrichment analysis and the terms “regulation of neuronal synaptic plasticity” and “learning” by the gene ontology analysis are upregulated in WT mice after original learning and in *Ccny* KO mice without training. **g**–**i** Learning-induced changes of actin-related molecules are affected in *Ccny* KO mice. **g** Heatmap of genes known to regulate spine morphology, *i.e*., those involved in the pathways of Rac, Cdc42, and Rho GTPases (listed on the right side of the heatmap). A change in the RNA expression levels was compared between WT and *Ccny* KO mice before training (Basal), after original learning (OL), and after original and reversal learning (RL). RNA expression levels of each gene are presented as the log_2_fold-change relative to Basal of WT. *n* = 3 mice for each sample group. **h** Fold-change of log_2_CPM for individual genes are plotted in WT and *Ccny* KO mice. *P < 0.05, **P < 0.005, ***P < 0.0005. **i** Genes whose transcript levels are increased in WT and further increased in *Ccny* KO mice (upper) or whose transcript levels are decreased in WT and further decreased in *Ccny* KO mice (lower) after original and reversal learning compared to those before training (Basal).

We found that the expression levels of *Ccny* mRNA detected by quantitative real-time polymerase chain reaction (qRT-PCR) (Fig. 4a) and CCNY protein detected by immunoblot analysis (Fig. 4b) were significantly reduced in the hippocampus after original learning in WT mice. Notably, improved spatial learning and memory ability in *Ccny* KO mice (Fig. 2b−f) and the decreased CCNY levels in WT mice following the original learning and memory task (Fig. 4a, b) together suggest a possible correlation between CCNY expression levels and cognitive function. Therefore, we aimed to determine whether *Ccny* KO mice in the naive state share molecular changes similar to those in WT mice subjected to original learning and memory. The potential candidates of molecular processes associated with original learning were examined by analyzing the transcriptome using high-throughput RNA sequencing. A total of 560 genes were differentially expressed when comparing WT mice under basal state with WT mice after original learning, in which 265 genes were upregulated and 295 genes were downregulated in the WT mice subjected to original learning (Fig. 4c; WT_Basal vs WT_OL). In untrained naive *Ccny* KO mice (KO_Basal), the expression levels of 314 genes were differentially regulated compared to untrained naive WT mice (WT_Basal), with 95 upregulated and 219 downregulated genes (Fig. 4c). Importantly, 29 genes that were commonly upregulated in both WT mice subjected to original learning and in untrained *Ccny* KO mice compared to WT mice under basal state (Fig. 4d, e) were significantly associated with the regulation of the actin cytoskeleton and the regulation of neuronal synaptic plasticity and learning, as revealed by the Kyoto Encyclopedia of Genes and Genomes enrichment analysis and the Gene Ontology analysis, respectively (Fig. 4f). These results strongly suggest that actin signaling plays a crucial role in mediating both the learning in WT mice and more improved learning ability in *Ccny* KO mice.

### Distinct expression of actin-related proteins induced by original and reversal learning in WT and *Ccny* KO mice

Next, we profiled dynamic changes in the expression levels of genes closely associated with actin reorganization and spine morphology, including molecular components in the pathways of Rac, Cdc42, and Rho GTPases, in the hippocampus of WT and *Ccny* KO mice before and after original and reversal learning and memory tasks (Fig. 4g). Without learning (Basal), the level of *Rac3* transcript was significantly higher in *Ccny* KO mice compared to WT mice, whereas the levels of *Acta2* and *Actb* transcripts were significantly lower in *Ccny* KO mice (Fig. 4hi). Original learning reduced the levels of *Rac3* and *Ssh3* transcripts in *Ccny* KO mice without significant changes in WT mice. In contrast, original learning decreased the levels of *Pak3* and *Wasl* transcripts in WT mice without significant changes in *Ccny* KO mice (Fig. 4hii). Reversal learning kept the reduction level of *Rac3* in *Ccny* KO mice but significantly decreased the *Rac3* level in WT mice when compared to original learning (Fig. 4hiii). Interestingly, changes in the *Ssh3* transcript levels were reversed by reversal learning in both WT and KO mice when compared to original learning (Fig. 4hiii). Original learning increased the levels of *Pak6*, *Limk1*, *Wasf3*, and *Acta1* transcripts in WT mice with no significant changes in *Ccny* KO mice (Fig. 4hii). The *Limk1* and *Wasf3* transcript levels remained unaltered after reversal learning versus original learning in both WT and *Ccny* KO mice, but the increase in the levels of *Pak6* and *Acta1* transcripts were greatly reduced after reversal learning in WT mice without significant changes in *Ccny* KO mice (Fig. 4hii−iv). These results together indicate that original and reversal learning processes are distinct at the molecular level and that such distinct molecular signaling is differentially modulated depending on the presence or absence of CCNY. Importantly, the expression of *Actb* transcripts increased after both original and reversal learning in WT mice, and *Ccny* KO mice exhibited more substantial increases in the *Actb* transcript level after original and reversal learning (Fig. 4hii−iv, i), highlighting the significance of the actin-mediated pathway underlying the improved original and reversal learning ability in *Ccny* KO mice, as observed in MWM behavioral tests (Figs. 2, 3).

### CCNY affects the LIMK1-cofilin-actin pathway distinctively in original and reversal learning

**Fig. 5:**
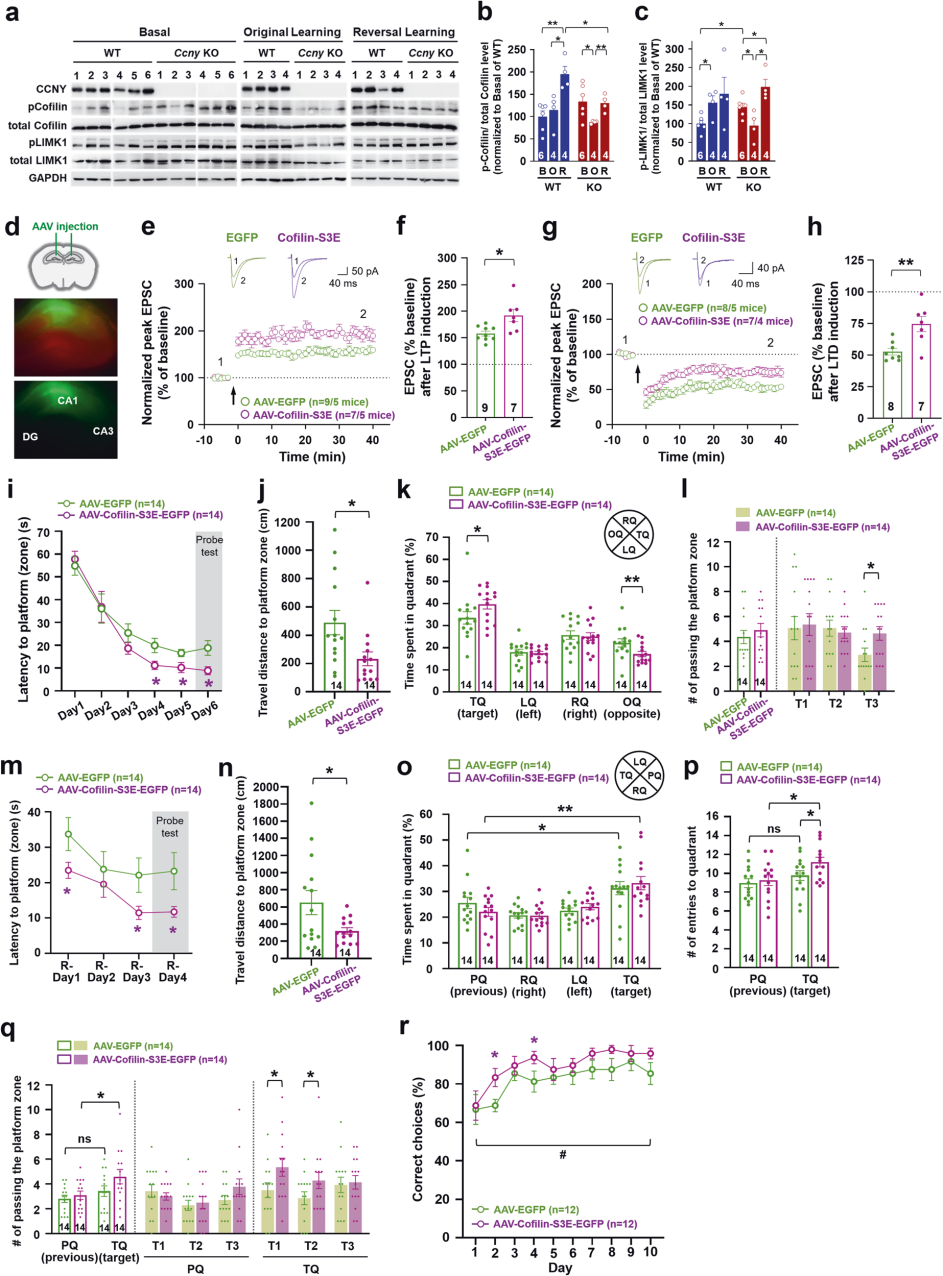
Increased phosphorylation of cofilin affects synaptic plasticity, spatial learning, and memory flexibility. **a**−**c** Original and reversal learning affects the LIM domain kinase 1 (LIMK1)-cofilin-actin pathway in *Ccny* KO mice distinctly from the WT mice. **a** Expression levels of the total and the phosphorylated form of cofilin and LIMK1 in the hippocampi from WT and *Ccny* KO mice after MWM original and reversal learning tasks were analyzed by immunoblotting. Full-length immunoblots are shown in Supplementary Fig. S8. **b**, **c** Ratio of the phosphorylated to total cofilin (**b**) and LIMK1 (**c**) protein expression levels in the hippocampi from WT and *Ccny* KO mice after the MWM original (O) and reversal learning (R) tasks. B, Basal; Data represent mean ± SEM. Data are normalized to the Basal of WT. *P < 0.05, **P < 0.005 as indicated, Student’s unpaired *t* test. **d** Hippocampal CA1 regions of WT mice were bilaterally injected with AAV overexpressing EGFP or a phosphomimetic mutant form of cofilin, cofilin-S3E-EGFP. The mice were subjected to LTP and LTD recordings, MWM task, and DNMTP T-maze task after 2 weeks (for LTP or LTD recording) or 30 days (for MWM or DNMTP T-maze task) following the viral injection. See also Supplementary Fig. S5 and S6. **e**, **f** Pairing-induced LTP is enhanced in mice overexpressing cofilin-S3E-EGFP in the CA1 region. **e** Data represent mean ± SEM of the peak EPSC amplitude relative to the baseline. LTP was induced by pairing stimulation (2 Hz for 90 s with 0 mV holding) at Schaffer collateral-CA1 synapses. *n* = 9 and 7 slices from 5 EGFP- and 5 cofilin-S3E-EGFP-overexpressing mice, respectively. Representative evoked EPSC traces before (1) and after (2) LTP induction are shown. Scale bars, 50 pA and 40 ms. **f** Data represent mean ± SEM of evoked EPSC amplitudes during the last 5 min (35−40 min) of the recordings in (**e**). *P < 0.05 as indicated, Student’s unpaired *t* test. **g**, **h** Pairing-induced LTD is reduced but still expressed in mice overexpressing cofilin-S3E-EGFP in the CA1 region. **g** Data represent mean ± SEM of the peak EPSC amplitude relative to the baseline. LTD was induced by a pairing protocol (5 Hz for 3 min with −40 mV holding) at Schaffer collateral-CA1 synapses. *n* = 8 and 7 slices from 5 EGFP- and 4 cofilin-S3E-EGFP-overexpressing mice, respectively. Representative evoked EPSC traces before (1) and after (2) LTD induction are shown. Scale bars, 40 pA and 40 ms. **h** Data represent mean ± SEM of evoked EPSC amplitudes during the last 5 min (35−40 min) of the recordings in (**g**). **P < 0.005 as indicated, Student’s unpaired *t* test. **i**–**k** Mice overexpressing cofilin-S3E-EGFP exh**i**bit improved spatial learning and memory. **i** Data represent mean ± SEM of the MWM learn**i**ng curve of latency to the platform during the original training sessions (Day 1 to 5) and latency to the platform zone during the probe test session (Day 6, gray shaded area). *P < 0.05, Student’s unpaired *t* test. **j** Data represent mean ± SEM of travel distance to the platform zone during the probe test on Day 6. *P < 0.05, Student’s unpaired *t* test. **k** Data represent mean ± SEM of the time spent in each quadrant during the probe test. TQ, target quadrant; LQ, quadrant left to the TQ; RQ, quadrant right to the TQ; OQ, quadrant opposite to the TQ. *P < 0.05, **P < 0.005 as indicated, Student’s unpaired *t* test. **l** The number of passing the platform zone during the original learning probe *t*est. CA1 regions of the hippocampi in wild-type mice were bilaterally injected with AAV overexpressing EGFP or Cofilin-S3E-EGFP. The mice were subjected to MWM 30 days after injection. TQ, target quadrant; LQ, quadrant left to the TQ; RQ, quadrant right to the TQ; OQ, quadrant opposite to the TQ. *P < 0.05 as indicated, Student’s unpaired *t* test. Empty bars are the merged data of T1, T2, and T3. **m**, **n** Mice overexpressing cofilin-S3E-EGFP show improved reversal learning and memory in the MWM task. **m** Data represent mean ± SEM of latency to the platform during the reversal learning training sessions (R-Day 1 to 3) and latency to the platform zone during the reversal learning probe test (R-Day 4, gray shaded area). *P < 0.05, Student’s unpaired *t* test. **n** Data represent mean ± SEM of travel distance to the platform zone during the reversal learning probe test. *P < 0.05, Student’s unpaired *t* test. **o** Time spent in each quadrant during the reversal learning probe test. Data represent mean ± SEM. PQ, previous *t*arget quadrant; TQ, target quadrant; LQ, quadrant left to the TQ; RQ, quadrant right to the TQ. *P < 0.05, **P < 0.005 as indicated, Student’s unpaired *t* test. **p** The number of entries to the previous target (PQ) and current target quadrant *(*TQ) during the reversal learning probe test. Data represent mean ± SEM. *P < 0.05, ns, not significant, as indicated, Student’s unpaired *t* test. **q** The number of passing the platform zone during the reversal learning probe test. *P < 0.05, ns, not significant, as indicated, Student’s unpaired *t* test. **r** Mice overexpressing cofilin-S3E-EGFP exhibit improved memory flexibility in a DNMTP T-maze task. Data represent mean ± SEM of the percentage of correct choices. *P < 0.05, Student’s unpaired *t* test. ^#^*P* < 0.05 as indicated, repeated-measures two-way ANOVA, the effect of genotype, F (1, 22) = 8.259. See also Supplementary Fig. S6.

Given that CCNY is an actin-binding protein and inhibits structural LTP by hindering the cofilin-actin pathway [26], we further investigated whether the improved original and reversal learning in *Ccny* KO (Figs. 2, 3) is associated with a change in the activity of cofilin, an actin-depolymerizing factor essential for actin reorganization [26, 48–52]. The levels of the phosphorylated inactive form of cofilin (p-cofilin) tended to increase after original learning in WT mice, but the p-cofilin level was significantly decreased in *Ccny* KO mice after original learning (Fig. 5a, b). Interestingly, *Ccny* KO mice without learning (basal) exhibited significantly higher level of the phosphorylated active form of LIMK1 (p-LIMK1), a kinase that phosphorylates cofilin, compared to the littermate WT mice, and also showed a tendency to increase the p-cofilin levels, albeit not significantly (Fig. 5b, c). Therefore, considering that *Ccny* KO mice under the basal state and WT mice subjected to original learning show comparable levels of p-LIMK1 and p-cofilin, it is likely that certain components of learning-induced molecular changes in the cofilin-actin pathway are already present in *Ccny* KO mice. This was supported by the finding that the actin cytoskeleton pathway was enriched by the set of genes that were commonly upregulated in WT mice after original learning and naive *Ccny* KO mice (Fig. 4f). In addition, reversal learning significantly increased p-cofilin levels in WT mice and rescued p-cofilin levels in *Ccny* KO mice to the level comparable to that observed in the basal condition (Fig. 5a, b). The level of p-LIMK1 was also altered similarly to the p-cofilin levels after original and reversal learning in both WT and *Ccny* KO mice (Fig. 5a, c), which was expected as LIMK1 is an upstream kinase for cofilin. In addition, the *Limk1* transcript level was increased after original learning in WT mice; however, this was not observed in *Ccny* KO mice (Fig. 4hii). Taken together, these results suggest that a decrease in the phosphorylation levels of cofilin (thus leading to elevated cofilin activity that facilitates actin depolymerization and enhances actin dynamics), and dynamic reversal of cofilin activity likely serve as the molecular basis for the improved original and reversal learning in *Ccny* KO mice, respectively (Fig. 5).

### The phosphorylation level of cofilin affects synaptic plasticity, spatial learning, and memory flexibility

Based on the RNA-seq data, we further determined whether the cofilin activity in *Ccny* KO mice is a critical factor that regulates synaptic plasticity, spatial learning and memory flexibility. We therefore investigated whether the increased p-cofilin level observed in *Ccny* KO mice at the basal state (Fig. 5a, b) is sufficient to modulate hippocampal synaptic plasticity, spatial learning, and memory flexibility and whether decreasing p-cofilin levels in *Ccny* KO mice could revert the observed altered synaptic plasticity and hippocampus-dependent learning. To test these possibilities, we generated another mouse model whose spectrum in synaptic response might be affected by increasing the phosphorylation levels of cofilin in WT mice as examined in *Ccny* KO mice through the stereotaxic injection of AAV expressing cofilin-S3E, a phosphomimetic mutant of cofilin, or by decreasing the phosphorylation levels of cofilin through the stereotaxic injection of AAV expressing cofilin-S3A, a non-phosphorylated active form of cofilin, into the CA1 region of *Ccny* KO mice (Fig. 5d). Intriguingly, viral-mediated overexpression of cofilin-S3E-EGFP reduced the expression levels of endogenous CCNY in the primary hippocampal neuron culture (Supplementary Fig. S5a, b) as well as in the CA1 region of hippocampal tissue (Supplementary Fig. S5d, e) compared to the controls. In addition, overexpression of phosphomimetic cofilin-S3E decreased the ratio of p-cofilin to the total levels of endogenous cofilin in the cultured hippocampal neurons (Supplementary Fig. S5a, c), as well as in the CA1 region of the hippocampus (Supplementary Fig. S5d, f). The AAV-mediated overexpression of cofilin-S3A-EGFP in the CA1 region increased the ratio of p-cofilin to the total levels of endogenous cofilin in WT (Supplementary Fig. S5g, i) and *Ccny* KO mice (Supplementary Fig. S5g, j) without affecting endogenous CCNY protein levels in WT mice (Supplementary Fig. S5g, h). The reduction and increase in the ratio of p-cofilin to the total endogenous cofilin levels observed upon the overexpression of cofilin-S3E and cofilin-S3A, respectively, might be caused by an intrinsic feedback mechanism required to maintain the total levels of p-cofilin, including endogenous p-cofilin and phosphomimetic cofilin-S3E or non-phosphorylatable cofilin-S3A, within appropriate ranges.

Similar to *Ccny* KO mice with increased p-cofilin levels (Fig. 5a, b), overexpression of cofilin-S3E-EGFP in the CA1 region significantly enhanced LTP (Fig. 5e, f) and reduced LTD (Fig. 5g, h) at Schaffer collateral-CA1 synapses in acute hippocampal slices compared to the control slices overexpressing EGFP in the CA1 region. In the original learning in the MWM task, the latency to the platform was significantly shorter on training days 4 and 5 in mice whose CA1 regions were infected with AAV-Cofilin-S3E-EGFP than in mice infected with AAV-EGFP (Fig. 5i). In addition, during the probe test on day 6, the latency (Fig. 5i) and travel distance (Fig. 5j) to the platform zone were significantly shorter in mice overexpressing cofilin-S3E-EGFP in the CA1 region. Even though the number of entries to the target quadrant was not significantly different between cofilin-S3E-EGFP-overexpressing and EGFP-overexpressing mice (Supplementary Fig. S6a), the time spent in the target quadrant was increased in mice overexpressing cofilin-S3E-EGFP compared to the mice overexpressing EGFP (Fig. 5k). The frequency of passing the platform zone tended to increase and was significantly increased in the third trial in mice overexpressing cofilin-S3E-EGFP compared to the control mice (Fig. 5l).

In a spatial reversal learning task, the latency to the platform was significantly shorter during the training sessions in mice overexpressing cofilin-S3E-EGFP compared to the control mice overexpressing EGFP (Fig. 5m). In addition, on day 4 of the reversal learning probe test, the latency (Fig. 5m) and travel distance (Fig. 5n) to the platform zone were also significantly reduced in mice overexpressing cofilin-S3E-EGFP. During the reversal learning probe test, mice overexpressing cofilin-S3E-EGFP spent more time in the target quadrant than in the previous target quadrant compared to the control mice overexpressing EGFP (Fig. 5o). The frequency of entries to the previous target quadrant relative to the current target quadrant (Fig. 5p) and the frequency of passing the previous target platform zone relative to the current target platform zone (Fig. 5q) were decreased in mice overexpressing cofilin-S3E-EGFP compared to the control mice. These results indicate that memory flexibility is improved in mice with enhanced phosphorylation levels of cofilin.

In agreement with the enhanced reversal learning (Fig. 5m−q), an improved learning flexibility in the DNMTP T-maze task was observed in mice overexpressing cofilin-S3E-EGFP (Fig. 5r). Both travel distance (Supplementary Fig. S6b) and travel time (Supplementary Fig. S6c) taken to make the correct choice in mice overexpressing cofilin-S3E-EGFP were comparable to those of the control mice. The body weights of the mice subjected to this task were maintained at approximately 80−87% of the initial body weight during the handling period throughout the experiments (Supplementary Fig. S6d).

**Fig. 6:**
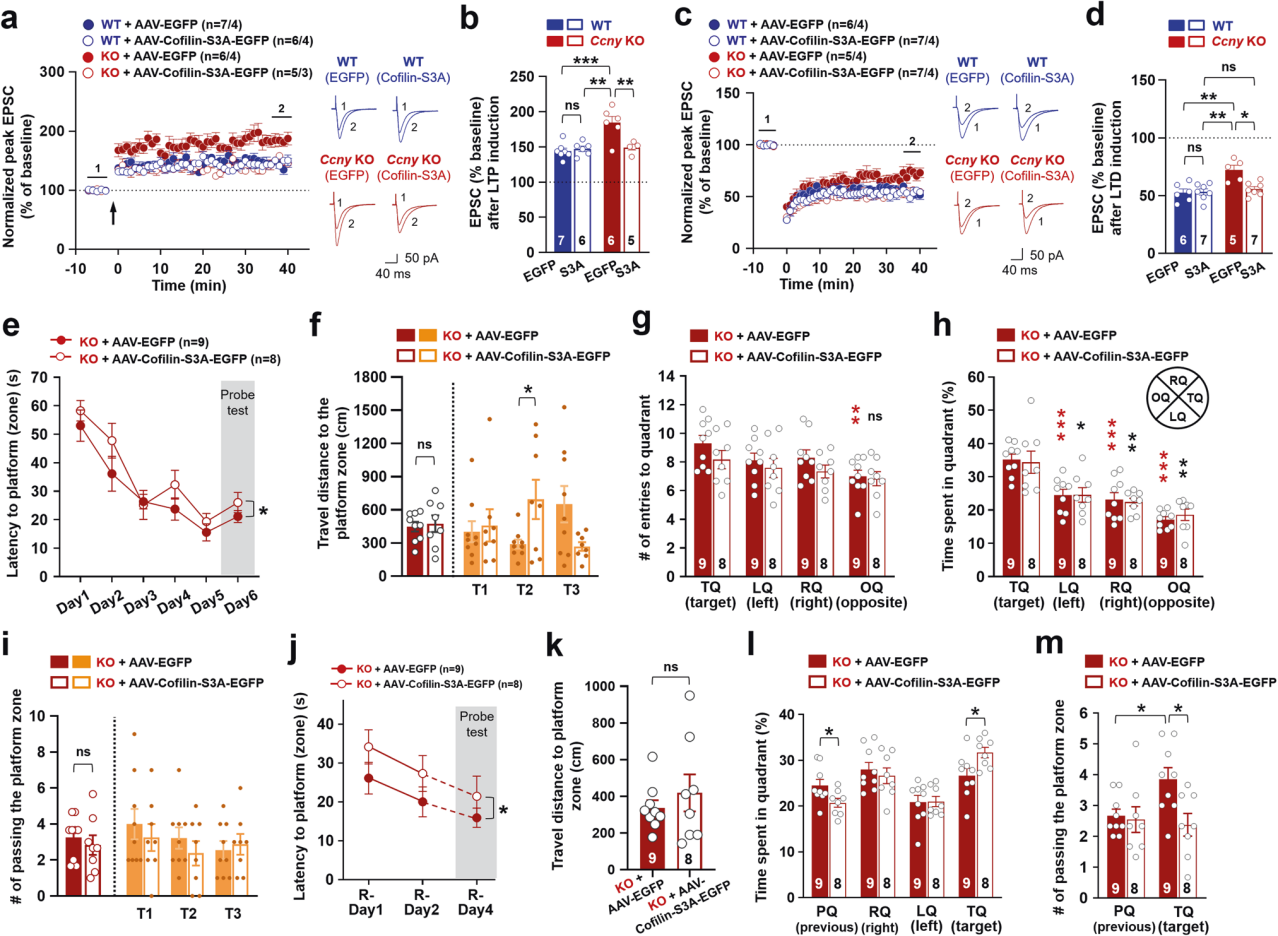
Decreasing the cofilin phosphorylation level in *Ccny* KO mice reverts the synaptic plasticity, spatial learning, and memory flexibility phenotypes observed in *Ccny* KO mice. **a**, **b** Enhanced LTP in *Ccny* KO mice is reverted to WT control levels with the overexpression of cofilin-S3A-EGFP in the CA1 region of *Ccny* KO mice. **a** Data represent mean ± SEM of the peak EPSC amplitude relative to the baseline. LTP was induced by pairing stimulation (2 Hz for 90 s with 0 mV holding) at Schaffer collateral-CA1 synapses. *n* = 7, 6, 6, and 5 slices from 4 EGFP-overexpressing WT, 4 cofilin-S3A-EGFP-overexpressing WT, 4 EGFP-overexpressing *Ccny* KO, and 3 cofilin-S3A-EGFP-overexpressing *Ccny* KO mice, respectively. Representative evoked EPSC traces before (1) and after (2) LTP induction are shown. Scale bars, 50 pA and 40 ms. **b** Data represent mean ± SEM of evoked EPSC amplitudes during the last 5 min (35–40 min) of the recordings in (**a**). **P < 0.005, ***P < 0.0005, ns, not significant as indicated, one-way ANOVA followed by Tukey’s multiple comparisons test. **c**, **d** Weakened LTD in *Ccny* KO mice is restored to the WT control level with the overexpression of cofilin-S3A-EGFP in the CA1 region of *Ccny* KO mice. **c** Data represent mean ± SEM of the peak EPSC amplitude relative to the baseline. LTD was induced by a pairing protocol (5 Hz for 3 min with −40 mV holding) at Schaffer collateral-CA1 synapses. *n* = 6, 7, 5, and 7 slices from 4 mice in each of 4 experimental groups. Representative evoked EPSC traces before (1) and after (2) LTD induction are shown. Scale bars, 50 pA and 40 ms. **d** Data represent mean ± SEM of evoked EPSC amplitudes during the last 5 min (35–40 min) of the recordings in (**c**). *P < 0.05, **P < 0.005, ns, not significant as indicated, one-way ANOVA followed by Tukey’s multiple comparisons test. **e**–**i** Overexpression of cofilin-S3A-EGFP in *Ccny* KO mice slightly but significantly reduces spatial learning and memory. **e** Learning curve of latency to platform (zone) during the MWM task. Data represent mean ± SEM. *P < 0.05 as indicated, two-way ANOVA. **f** Travel distance to the platform zone during the probe test. ns, not significant, *P < 0.05 as indicated, Student’s unpaired *t* test. Bars on the left side of the dotted line are the averaged data of T1, T2, and T3. **g**, **h** Data represent mean ± SEM of the number of entries to (**g**) and time spent in (**h**) each quadrant during the probe test on Day 6. ns, not significant, *P < 0.05, **P < 0.005, ***P < 0.0005 compared to the TQ, Student’s unpaired *t* test. (i) The number of passing the platform zone during the probe test. ns, not significant as indicated, Student’s unpaired *t* test. **j**−**m** Overexpression of cofilin-S3A-EGFP in *Ccny* KO mice reduces memory flexibility in reversal learning of the MWM task. **j** Latency to platform (zone) during the reversal learning. Data represent mean ± SEM. *P < 0.05 as indicated, two-way ANOVA. **k** Travel distance to the platform zone during the reversal learning probe test. ns, not significant, Student’s unpaired *t* test. (l) Time spent in each quadrant during the reversal learning probe test. Data represent mean ± SEM. *P < 0.05 as indicated, Student’s unpaired *t* test. **m** The number of passing the platform zone during the reversal learning probe test. Data represent mean ± SEM. *P < 0.05 as indicated, Student’s unpaired *t* test. See also Supplementary Fig. S5.

We next examined whether overexpression of the non-phosphorylatable form of cofilin would reduce the synaptic and learning behavioral phenotypes of *Ccny* KO mice. Overexpression of cofilin-S3A-EGFP in the CA1 region did not affect LTP (Fig. 6a, b) [53] and LTD in WT littermates (Fig. 6c, d), whereas the overexpression of cofilin-S3A-EGFP in *Ccny* KO mice reverted the enhanced LTP (Fig. 6a, b) and reduced LTD (Fig. 6c, d) that were observed in *Ccny* KO mice to levels similar to those observed in WT littermates overexpressing EGFP in the CA1 region. In agreement with LTP results, the overexpression of cofilin-S3A-EGFP in *Ccny* KO mice slightly but significantly increased latency time to arrive at the platform (zone) during the original learning period in the MWM task (Fig. 6e) compared to that observed with EGFP-overexpressing control *Ccny* KO mice. Even though time spent in the quadrant was not altered in *Ccny* KO mice overexpressing cofilin-S3A-EGFP (Fig. 6h), travel distance to the platform zone tended to increase in the first trial (T1) and significantly increased in the second trial (T2) compared to that observed with EGFP-overexpressing control *Ccny* KO mice (Fig. 6f). The number of entries to the target quadrant (Fig. 6g) and the frequency of passing the platform zone in T1 and T2 (Fig. 6i) also tended to decrease with cofilin-S3A-EGFP-overexpressing *Ccny* KO mice compared to EGFP-overexpressing *Ccny* KO mice. Furthermore, the shortened latency to the platform (zone) during the reversal learning in the MWM task observed with *Ccny* KO mice reverted with the overexpression of cofilin-S3A-EGFP (Fig. 6j). Even though *Ccny* KO mice overexpressing cofilin-S3A-EGFP spent less time in the previous quadrant and more time in the target quadrant (Fig. 6l), those mice tended to show a longer travel distance (Fig. 6k) and further showed a significant reduction in the frequency of passing the target quadrant (Fig. 6m).

In summary, these results indicate that the phosphorylation levels of cofilin are sufficient to modulate synaptic plasticity, spatial learning, and memory flexibility in *Ccny* KO mice. In addition, the data from *Ccny* KO, cofilin-S3E-EGFP-overexpressing, or cofilin-S3A-EGFP-overexpressing mice indicate that CCNY expression level and cofilin phosphorylation signaling reciprocally affect each other, which ultimately regulates synaptic plasticity, spatial learning, and memory flexibility. Furthermore, all the data from *Ccny* KO, *Ccny* KO treated with Ro25-6981, GluN2B-C456Y-mutant, or cofilin-S3E-EGFP-overexpressing mice indicate that memory flexibility does not necessarily require intact LTD, but reduced LTD (examined in *Ccny* KO, GluN2B-C456Y-mutant, or cofilin-S3E-EGFP-overexpressing mouse models) supports memory flexibility when combined with normal LTP/spatial learning (as in GluN2B-C456Y-mutant mice) or enhanced LTP/improved spatial learning (as in *Ccny* KO or cofilin-S3E-EGFP-overexpressing mice). Therefore, CCNY is proposed as a learning regulator that modulates both memorizing (in the processes of both spatial learning and memory flexibility) and forgetting (in the process of memory flexibility) processes.

## Discussion

Here, we demonstrate that reduced LTD, but not necessarily intact LTD, is able to mediate memory flexibility when LTP is intact or enhanced, suggesting a possible intercommunication between LTP and LTD in the context of new learning and existing memory weakening during memory flexibility (Supplementary Fig. S7①). In addition, the expression levels of genes associated with actin reorganization were differentially regulated by original and reversal learning in a CCNY-dependent manner (Fig. 4g−i). Both original (spatial learning, Supplementary Fig. S7, red line) and reversal learning (memory flexibility, Supplementary Fig. S7, blue line) increased p-LIMK1 and p-cofilin levels (Fig. 5). Interestingly, CCNY facilitated phosphorylation of LIMK1 (active) and cofilin (inactive) following original learning (Fig. 5; Supplementary Fig. S7②), whereas it inhibited the phosphorylation of LIMK1 and cofilin following reversal learning (Fig. 5; Supplementary Fig. S7③), indicating that spatial learning and memory flexibility are distinctively modulated by the LIMK1-cofilin-actin pathway in a CCNY-dependent manner. Furthermore, increasing the phosphorylation level of cofilin, as observed in the *Ccny* KO mice, in the CA1 regions of the hippocampi by the transduction of AAV overexpressing cofilin-S3E plays a causative role in the changes observed in synaptic plasticity, including enhancement of LTP (Fig. 5e, f; Supplementary Fig. S7④, black thin arrow) and reduction of LTD (Fig. 5g, h; Supplementary Fig. S7⑤, black dotted line), as well as for the enhancement of spatial learning (Fig. 5i−l; Supplementary Fig. S7④, black thick arrow) and memory flexibility (Fig. 5m−r; Supplementary Fig. S7⑤, black thick arrow). The present study also suggests that CCNY is a learning regulator that modulates both the memorizing and forgetting processes. Further studies will be needed to determine how cofilin phosphorylation levels are regulated differently in the presence or absence of CCNY in the context of synaptic plasticity and cognitive functions such as spatial learning and memory flexibility.

LTP serves as a cellular mechanism underlying learning and memory formation in the brain and is bi-directionally modulated by enhancers and suppressors. LTD is also required for spatial reversal learning and memory (i.e., memory flexibility) [3–6, 54], and learning per se facilitates LTD [7–12]. Memory extinction is a process that involves new learning that suppresses the expression of prior memory [55], and importantly, both the time spent in the previous target quadrant (Fig. 2k) and the frequency of entries to the previous target quadrant (Fig. 2l) during the probe test after reversal learning were significantly deceased in *Ccny* KO mice, thereby implying the facilitated extinction of the initial spatial memory in *Ccny* KO mice. This facilitated memory extinction, a weakening of the previously acquired memory information required for new learning or memory flexibility, could be mediated by LTD expressed in *Ccny* KO mice (Fig. 1l, m). Importantly, however, the magnitude of LTD expression was reduced in *Ccny* KO mice compared to WT littermates (Fig. 1l, m), suggesting that modest LTD, but not necessarily strongly expressed LTD, could be sufficient for weakening the existing memory. Moreover, considering that memory flexibility also involves new learning in addition to the extinction of prior memory, and that learning is improved in *Ccny* KO mice, it is possible that both LTD and LTP play a critical role in facilitating memory flexibility.

Even though a complete blockade of LTD (but with normal spatial learning and memory ability) was reported to be associated with impaired memory flexibility [3, 27], our observations indicate that even weak LTD, together with enhanced spatial learning and memory ability, can exhibit memory flexibility. These findings add more mechanistic insights into the current simple view of the relationship between LTD and memory flexibility. In memory flexibility, which comprises new learning and a weakening of previous memory, new learning may be mediated by LTP, and a weakening of previous memory may be mediated by LTD. Our results suggest that these two processes of new learning and weakening of previous memory are mechanistically interconnected and that the precise tuning of these two processes can determine the degree of memory flexibility. Along with the findings of the present study, several previous studies support this notion. Double KO mice lacking both type 1 and 8 adenylyl cyclases show defective LTP and LTD, along with a deficit in both original and reversal learning [56]. In addition, mice overexpressing type 1 adenylyl cyclase exhibit enhanced LTP and impaired LTD at the Schaffer collateral-CA1 synapses and show superior original as well as reversal learning [57]. Therefore, in some instances, memory flexibility can be exhibited by the combination of enhanced LTP and reduced LTD. Considering that *Ccny* KO mice with enhanced LTP and moderate LTD show superior original and reversal learning, it is likely that enhanced LTP improves certain aspects of memory flexibility, presumably by facilitating new memory formation, which may compensate for the weakened LTD-derived ineffective extinction of the previously established memory.

Memory formation should be adequately regulated within physiological ranges, which requires maintaining the balance between memorizing (acquisition and consolidation) and forgetting (extinction) processes. Learning and memory have been extensively investigated in terms of acquisition and consolidation [13, 58–61], and the extinction and reconsolidation processes have also received attention in the field of learning and memory [14, 62–64]. Failure to maintain physiological memory strength may cause pathological malfunctioning of learning and memory, such as exhibition of weak or strong memory beyond the physiological range, resulting in deficits in learning and memory flexibility. In the present study, we demonstrate that CCNY plays a pivotal role in both spatial learning and memory flexibility, and *Ccny* KO mice will be useful as learning and memory research models to investigate the brain circuitry involved in spatial learning and memory flexibility in physiology and pathology.

## Materials and methods

All materials and methods, except animals and data availability, are included in the Supplementary information. A summary of the statistical analysis in each figure is provided in Supplementary Table S3.

### Animals

All experiments involving animals were performed in accordance with the guidelines and regulations of the Korea Institute of Science and Technology (KIST) or Korea Advanced Institute of Science and Technology (KAIST). All experimental protocols were approved by the KIST or KAIST Institutional Animal Care and Use Committee (approval number 2019-054 at KIST; KA2012-19 at KAIST).

## Supplementary information

Supplementary Figures S1-S8

Supplementary Materials and methods

Supplementary Table S1

Supplementary Table S2

Supplementary Table S3

## Data Availability

RNA-seq data were deposited in the National Center for Biotechnology Information (NCBI) Gene Expression Omnibus (GEO) and the Korean Nucleotide Archive (KoNA) under the accession numbers GSE129324 and PRJKA220513, respectively.
