# Supplementary material for: Cyclin Y regulates spatial learning and memory flexibility through distinct control of the actin pathway
Source: Mol Psychiatry. 2022 Nov 25;28(3):1351–64. doi: 10.1038/s41380-022-01877-0 (PMC10005959; doi:10.1038/s41380-022-01877-0)
Supplement: Supplementary file 1 — Supplementary Figures S1-S8 [file 41380_2022_1877_MOESM1_ESM.pdf]

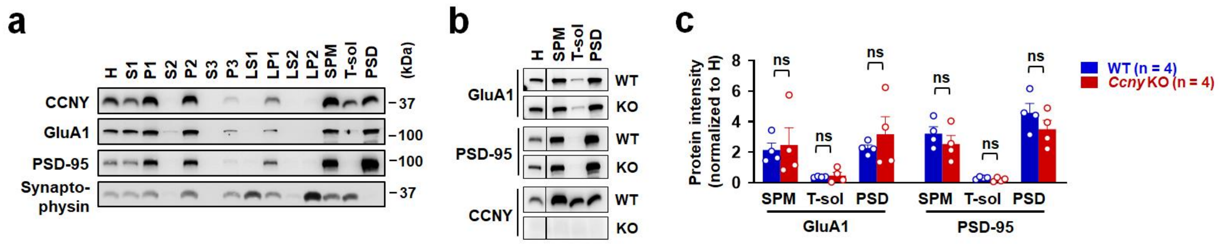

Supplementary Fig. S1. CCNY and excitatory synaptic protein expression levels in wild-type (WT) and *Ccny* knockout (KO) mice. (a) Endogenous CCNY is located in postsynaptic fractions in the mouse brain. Forebrain samples of male mice (18 weeks old) were used for subcellular fractionation, and a total of 5 μg protein of each fraction was examined in immunoblotting experiments. H, homogenates; P1, nuclear pellet; P2, crude synaptosomal fraction; S3, cytosolic fraction; LP1, synaptosomal membrane fraction; LP2, synaptic vesicle-enriched fraction; SPM, synaptic plasma membrane fraction; T-sol, Tx-100-soluble fraction; PSD, postsynaptic density fraction. Full-length immunoblots are shown in Supplementary Fig. S8. **(b, c)** Synaptic localizations of GluA1 and PSD-95 are not altered in the *Ccny* KO forebrain. H, homogenates; SPM, synaptic plasma membrane fraction; T-sol, Tx-100-soluble fraction; PSD, postsynaptic density fraction; ns, not significant as indicated, Student's unpaired t test. Full-length immunoblots are shown in Supplementary Fig. S8.

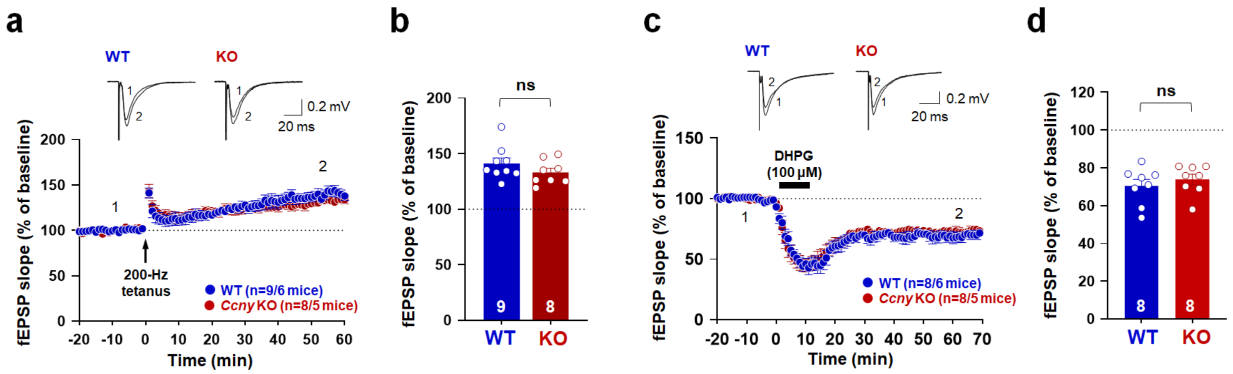

Supplementary Fig. S2. NMDAR-independent forms of synaptic plasticity are not affected in *Ccny* KO mice. (a, b) Voltage-gated calcium channel (VGCC)-dependent LTP at Schaffer collateral-CA1 synapses is not altered in *Ccny* KO mice. **(a)** Data represent mean ± SEM of the fEPSP slope relative to the baseline. All recordings were made in the presence of 50 μM D-APV to block NMDARs. VGCC-dependent LTP was induced by 4 stimulus trains (200 Hz, 0.5 s) delivered at a rate of 1 train per 5 s. n = 9 and 8 slices from 6 WT and 5 KO mice, respectively. Representative fEPSP traces from WT and KO mice before (1) and after (2) LTP induction. Scale bars, 0.2 mV and 20 ms. **(b)** Data represent mean ± SEM of fEPSP slopes during the last 10 min (50–60 min) of the recordings in (**a**). ns, not significant, Student's unpaired *t* test. **(c, d)** Metabotropic glutamate receptor (mGluR)-dependent LTD at Schaffer collateral-CA1 synapses is not altered in *Ccny* KO mice. **(c)** Data represent mean ± SEM of the fEPSP slope relative to the baseline. All recordings were made in the presence of 50 μM picrotoxin and 5 μM L-689,560. mGluR-dependent LTD was induced by the application of 100 μM DHPG for 10 min. n = 8 and 8 slices from 6 WT and 5 KO mice, respectively. Representative fEPSP traces from WT and KO mice before (1) and after (2) LTD induction. Scale bars, 0.2 mV and 20 ms. **(d)** Data represent mean ± SEM of fEPSP slopes during the last 10 min (60–70 min) of the recordings in (**c**). ns, not significant, Student's unpaired *t* test.

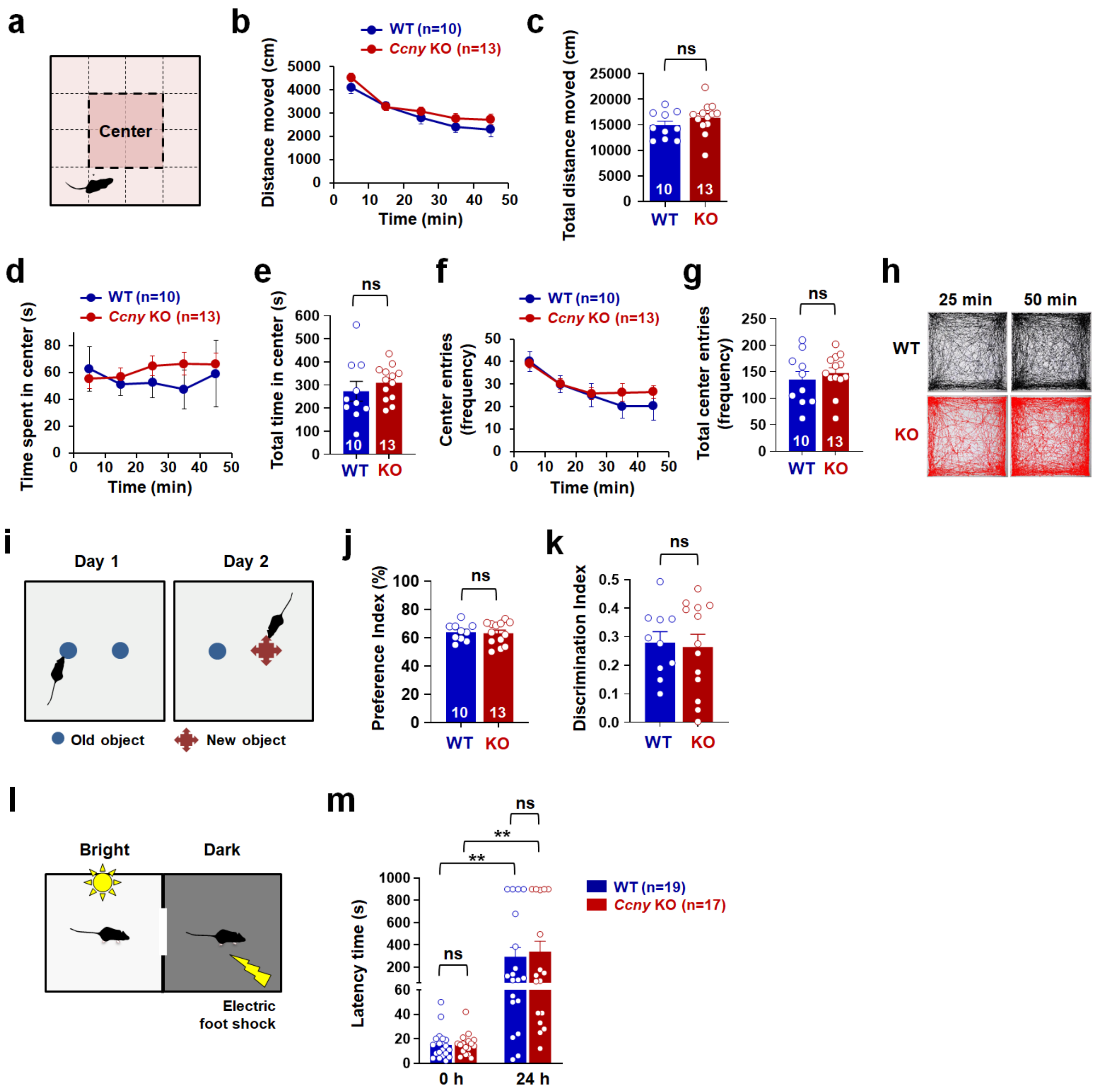

Supplementary Fig. S3. Animal behaviors in open field, novel object recognition, and passive avoidance tasks are not affected in *Ccny* KO mice. (a) Schematic diagram for the open field test. The region for "center" was defined as a square within 20 cm in the center of the box as shown in the diagram. **(b–h)** Distance moved during every 10 min (**b**) and for the total 50-min duration (**c**), time spent in center every 10 min (**d**) and for the total 50-min duration (**e**), and the frequency of center entries every 10 min (**f**) and for the total 50-min duration (**g**) were measured. Trajectories for the mouse activities in the open field are shown for the first 25 and the

total 50 min (**h**). Data represent mean ± SEM. ns, not significant as indicated, $P = 0.120$ (**c**), $P = 0.208$ (**e**), and $P = 0.251$ (**g**), Student's unpaired *t* test. Results obtained from two cohorts. **(i)** Schematic diagram for the novel object recognition task. **(j, k)** No difference in the novel object recognition task between WT and *Ccny* KO mice. The preference index (**j**) was calculated as the percentage of time spent exploring the novel object divided by the total time spent exploring the old or novel object for 10 min. The discrimination index (**k**) was calculated as follows: the time spent exploring the old object is subtracted from the time spent exploring the novel object, which is then divided by the total time spent exploring the old or the novel object for 10 min. Data represent mean ± SEM. ns, not significant as indicated, $P = 0.396$ (**j**) and $P = 0.395$ (**k**), Student's unpaired *t* test. Data obtained from two cohorts. **(l)** Schematic diagram for the passive avoidance task. **(m)** Latency time to enter the dark chamber was measured 24 h after the training session during which an electric foot-shock (0.35 mA, 1 s) was delivered once the mouse entered the black chamber from the brightly lit chamber. Data represent mean ± SEM. ns, not significant, $^{**}P < 0.005$ as indicated, $P = 0.456$ (0 h; WT vs KO), $P = 0.359$ (24 h; WT vs KO), $P = 0.0008$ (WT; 0 h vs 24 h), $P = 0.0008$ (KO; 0 h vs 24 h), Student's unpaired *t* test. Data obtained from two cohorts.

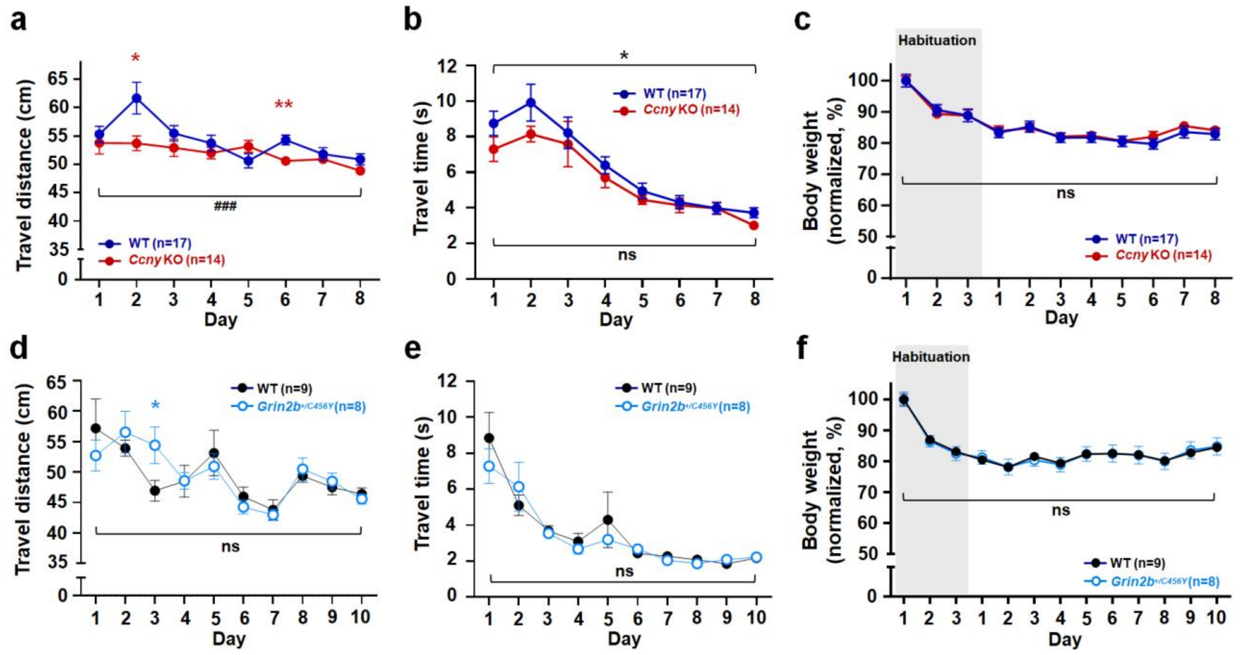

Supplementary Fig. S4. Additional data for the delayed nonmatch to place T-maze task performed for Figure 2n and o. (a) WT and *Ccny* KO mice were applied to delayed nonmatch to place T-maze task. Data represent mean ± SEM of travel distance during the choice run. *P < 0.05, **P < 0.005, P = 0.011 (Day 2, WT vs *Ccny* KO) and P = 0.002 (Day 6, WT vs *Ccny* KO), Student's unpaired t test. $^{\#\#\#}P$ < 0.0005, $^{\#\#\#}P$ = 0.00048, repeated-measures two-way ANOVA, the effect of genotype, F (1, 29) = 8.177. **(b)** Data represent mean ± SEM of travel time during the choice run. *P < 0.05, P = 0.025 (paired by day over 8 days), Student's paired t test. ns, not significant, P = 0.161, repeated-measures two-way ANOVA, the effect of genotype, F (1, 29) = 2.067. **(c)** Data represent mean ± SEM of body weight. ns, not significant, P = 0.163 (paired by day over 8 days), Student's paired t test. **(d)** Data represent mean ± SEM of travel distance during the choice run. *P < 0.05, P = 0.005 (Day 3, WT vs *Grin2b*$^{+/C456Y}$), Student's unpaired t test. ns, not significant, P = 0.921, repeated-measures two-way ANOVA, the effect of genotype, F (1, 15) = 0.010. **(e)** Data represent mean ± SEM of travel time during the choice run. P = 0.378 (paired by day over 10 days), Student's paired t test. ns, not significant, P = 0.561, repeated-measures two-way ANOVA, the effect of genotype, F (1, 15) = 0.353. **(f)** Data represent mean ± SEM of body weight. ns, not significant, P = 0.552 (paired by day over 13 days), Student's paired t test.

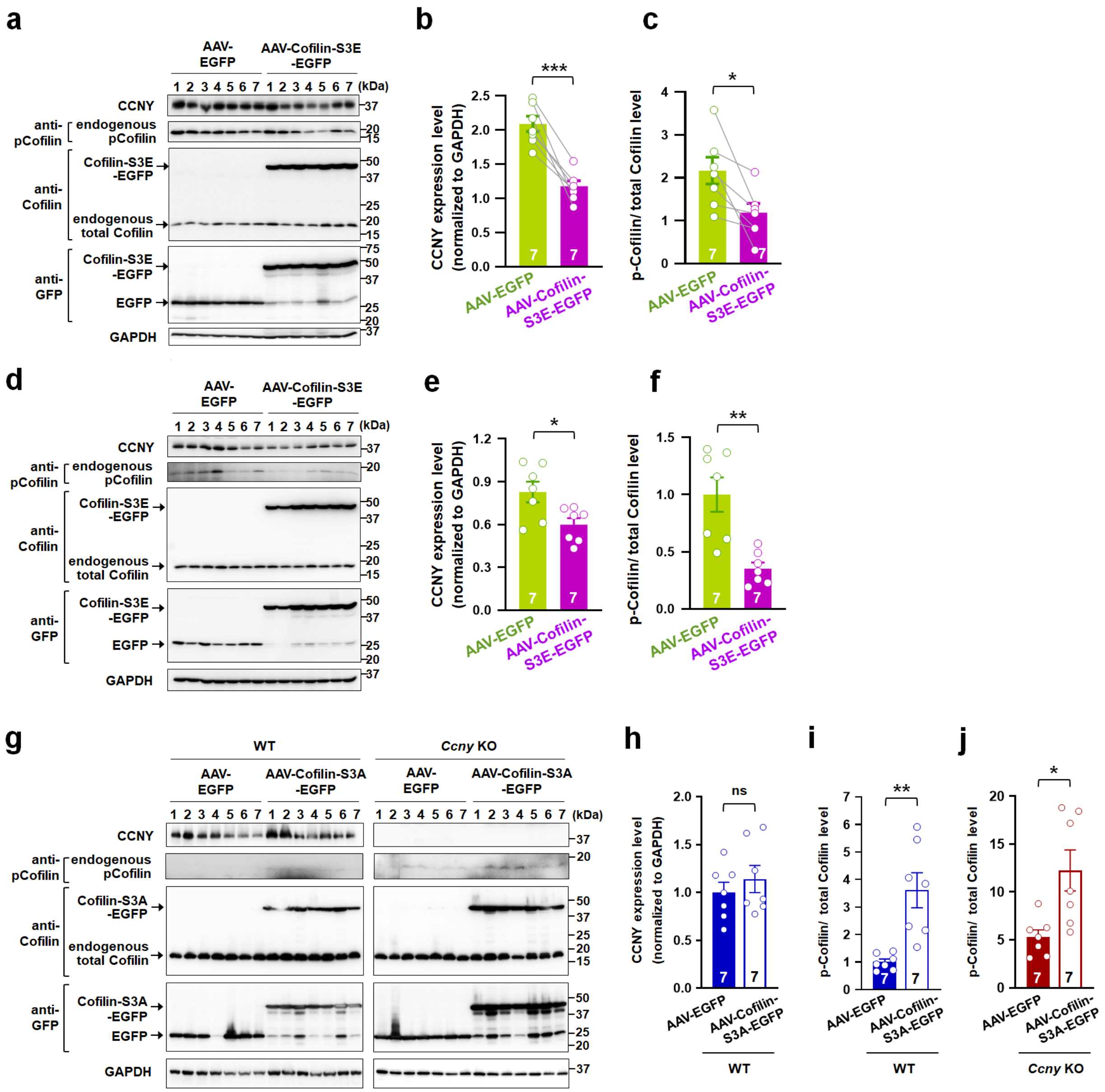

Supplementary Fig. S5. CCNY expression level and the ratio of phosphorylated to total cofilin protein expression levels in neurons overexpressing cofilin-S3E, a phosphomimetic mutant, or cofilin-S3A, a non-phosphorylated mutant. (a–f) Overexpression of cofilin-S3E decreases the CCNY expression level and the ratio of phosphorylated to total cofilin protein expression levels. Immunoblot analysis showing the expression levels of endogenous CCNY and the total and phosphorylated forms of endogenous cofilin in cultured hippocampal neurons (**a**) or in the dorsal CA1 region of the hippocampus (**d**) infected with AAV expressing EGFP (AAV-

EGFP) or cofilin-S3E (AAV-Cofilin-S3E-EGFP). AAV were infected at DIV 5 and expressed for 14 days (**a**) or injected into the CA1 regions at P28-32 and expressed for 30 days (**d**). **(b, e)** CCNY protein expression levels were analyzed from (**a**) and (**d**). Data are normalized to GAPDH expression levels. **(c, f)** The ratio of phosphorylated to total cofilin protein expression levels was analyzed from (**a**) and (**d**). Data represent mean ± SEM. ***P = 0.0003 in (**b**), *P = 0.018 in (**c**), Student's paired t test. *P = 0.020 in (**e**), **P = 0.0015 in (**f**), Student's unpaired t test. **(g–j)** Overexpression of cofilin-S3A increases the ratio of phosphorylated to total cofilin protein expression levels but does not affect the CCNY expression level. Immunoblot analysis showing the expression levels of endogenous CCNY and the total and phosphorylated forms of endogenous cofilin in the dorsal CA1 region of the hippocampus (**g**) infected with AAV expressing EGFP (AAV-EGFP) or cofilin-S3A (AAV-Cofilin-S3A-EGFP). AAV were injected into the CA1 regions at P28-32 and expressed for 30 days. **(h)** CCNY protein expression levels were analyzed from (**g**). Data are normalized to GAPDH expression levels. **(j)** The ratio of phosphorylated to total cofilin protein expression levels was analyzed from (**g**). Data represent mean ± SEM. P = 0.4482 in (**h**), **P = 0.0016 in (**i**), *P = 0.0101 in (**j**), ns, not significant, Student's unpaired t test. Full-length immunoblots are shown in Supplementary Fig. S8.

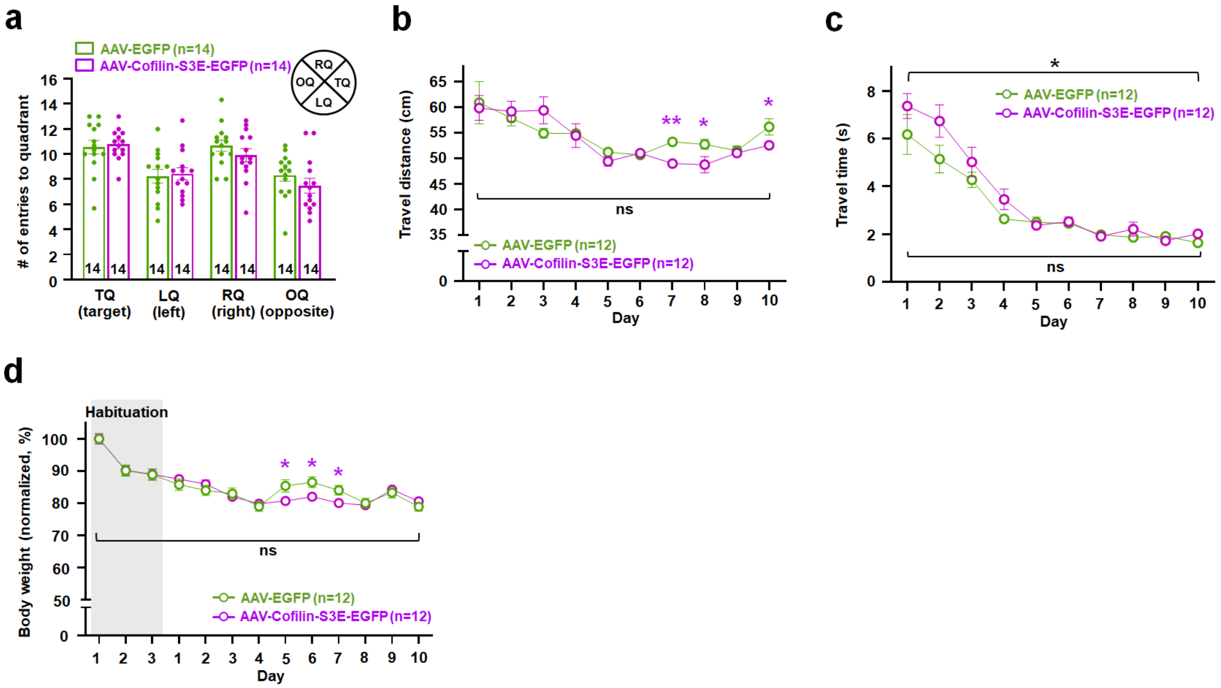

Supplementary Fig. S6. Additional data for Morris water maze (MWM) and a delayed nonmatch to place T-maze tasks performed for Figure 5. (a) The number of entries to each quadrant during the original learning probe test. CA1 regions of hippocampi in wild-type mice were bilaterally injected with AAV overexpressing EGFP or Cofilin-S3E-EGFP. The mice were subjected to the Morris Water Maze (MWM) 30 days after the injection. TQ, target quadrant; LQ, quadrant left to the TQ; RQ, quadrant right to the TQ; OQ, quadrant opposite to the TQ. $*P < 0.05$ as indicated, $P = 0.337$ (TQ), $P = 0.367$ (LQ), $P = 0.106$ (RQ), and $P = 0.060$ (OQ), Student's unpaired t test. **(b)** Data represent mean ± SEM of travel distance during the choice run. $*P < 0.05$, $**P < 0.005$, $P = 0.00076$ (Day 7), $P = 0.045$ (Day 8), and $P = 0.043$ (Day 10), Student's unpaired t test. ns, not significant, $P = 0.219$, repeated-measures two-way ANOVA, the effect of genotype, F (1, 22) = 1.596. **(c)** Data represent mean ± SEM of travel time during the choice run. $P = 0.033$ (paired by day over 10 days), Student's paired t test. ns, not significant, $P = 0.058$, repeated-measures two-way ANOVA, the effect of genotype, F (1, 22) = 3.989. **(d)** Data represent mean ± SEM of body weight. $*P < 0.05$, $P = 0.023$ (Day 5), $P = 0.019$ (Day 6), $P = 0.021$ (Day 7), Student's unpaired t test. ns, not significant, $P = 0.403$ (paired by day over 13 days), Student's paired t test.

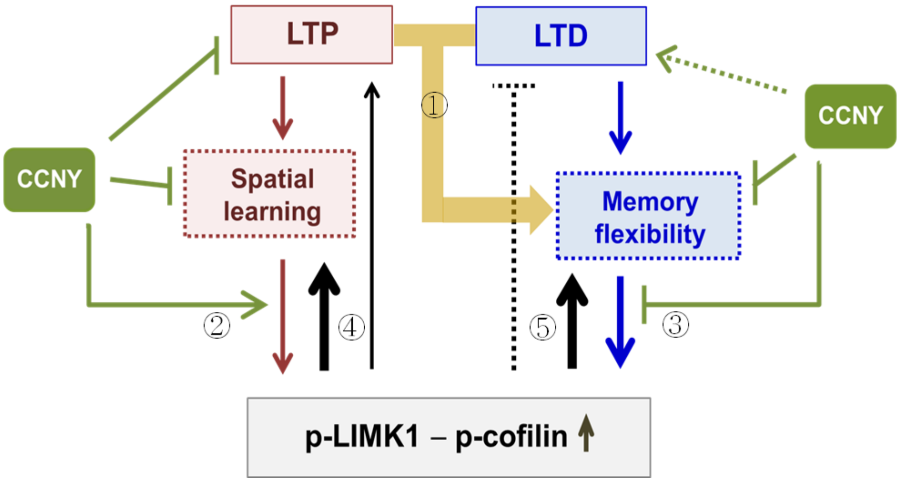

Supplementary Fig. S7. Summary for distinct regulations of the LIMK1-cofilin signaling pathway and synaptic plasticity in the context of spatial learning and memory flexibility. See the main text for details.

Fig. 5a

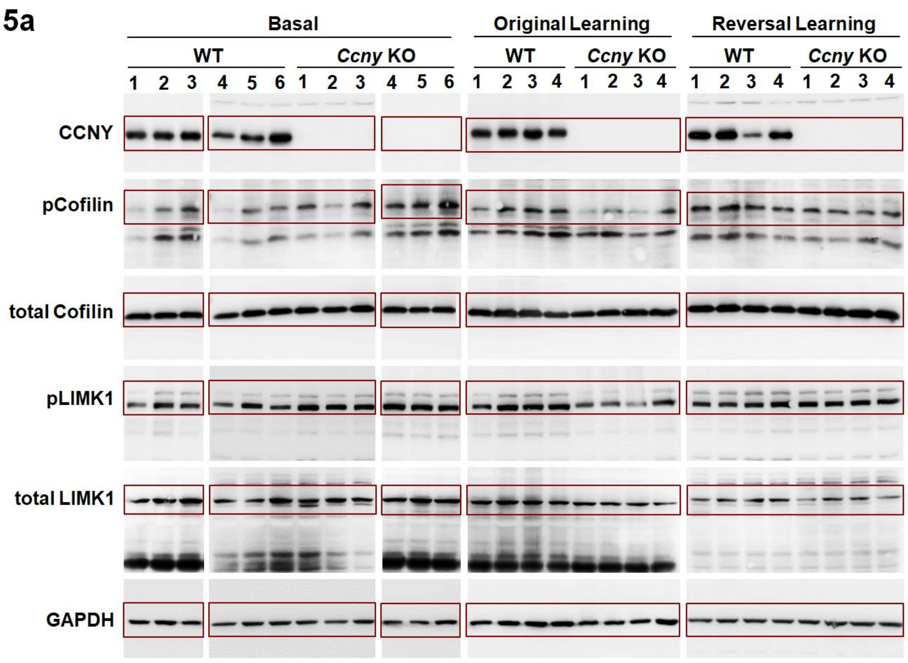

Fig. S1a

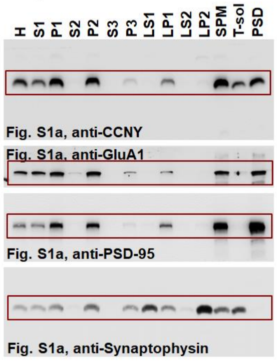

Fig. S1b

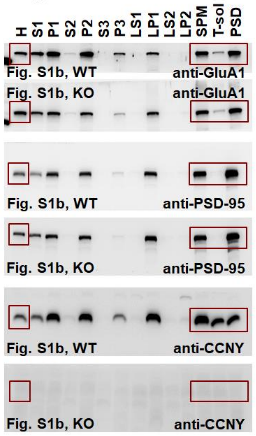

Fig. S5a

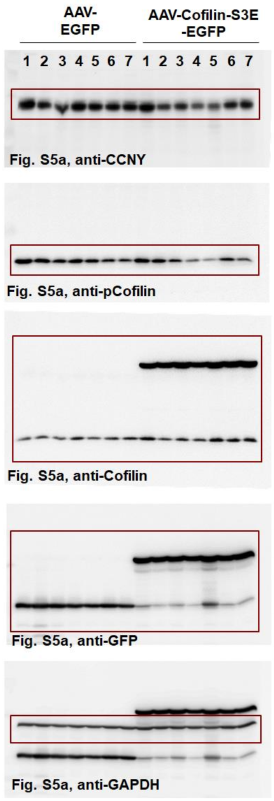

Fig. S5d

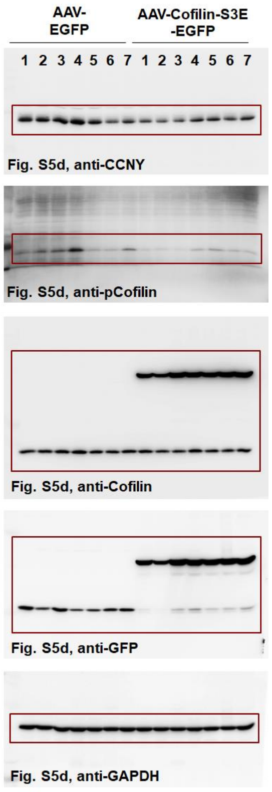

Fig. S5g

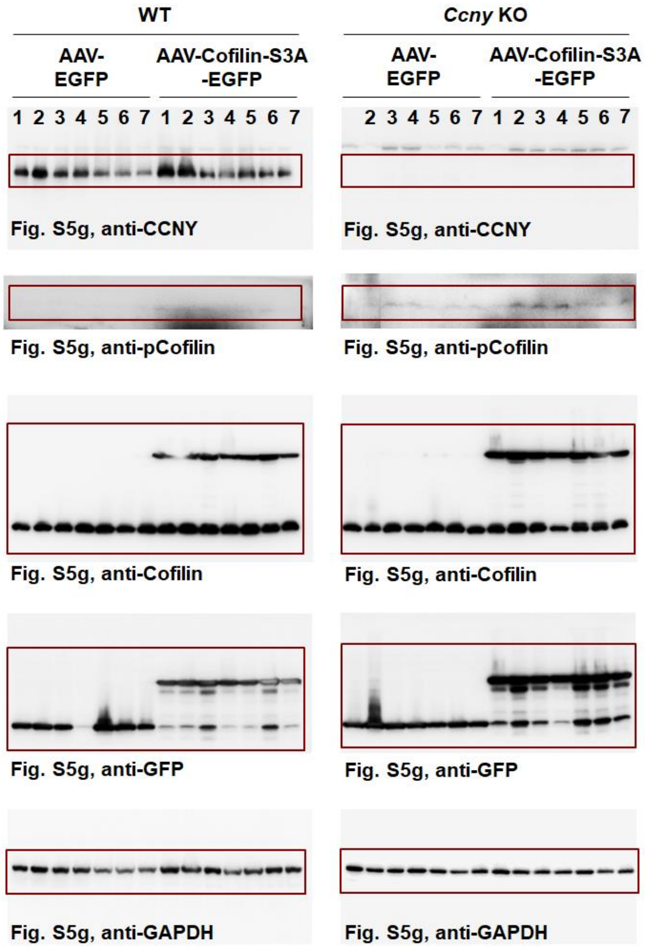

Supplementary Fig. S8. Full-length immunoblots used in this study. Red square boxes indicate the cropped areas presented in Figure 5a, S1a, S1b, S5a, S5d, and S5g.
