# Supplementary material for: Cyclin Y regulates spatial learning and memory flexibility through distinct control of the actin pathway
Source: Mol Psychiatry. 2022 Nov 25;28(3):1351–64. doi: 10.1038/s41380-022-01877-0 (PMC10005959; doi:10.1038/s41380-022-01877-0)
Supplement: Supplementary file 2 — Supplementary Materials and methods [file 41380_2022_1877_MOESM2_ESM.doc]

**Supplementary Information**

**Supplementary Materials and methods…...................................................................................2**

**Supplementary References…......................................................................................................13**

**Supplementary Materials and methods**

**DNA constructs**

To construct adeno-associated virus (AAV) vectors expressing human cofilin-S3E-EGFP (pAAV-cofilin-S3E-EGFP) or human cofilin-S3A-EGFP (pAAV-cofilin-S3A-EGFP), the inserts containing the human cofilin-S3E-EGFP or cofilin-S3A-EGFP fragment was obtained by digesting pEGFP-N1-human cofilin-S3E or pEGFP-N1-human cofilin-S3A with EcoRI/HpaI restriction enzymes, and the insert was then subcloned into pAAV-MCS expression vector (VPK-410, Cell Biolabs, Inc.) digested with EcoRI/HincII restriction enzymes. pAAV-EGFP was a gift from the KIST Virus Facility ([http://virus.kist.re.kr](http://virus.kist.re.kr/)). pEGFP-N1-human cofilin-S3E (Addgene plasmid #50861; http://n2t.net/addgene:50861; RRID:Addgene_50861) and pEGFP-N1-human cofilin-S3A (Addgene plasmid #50860; http://n2t.net/addgene:50860; RRID:Addgene_50860) were gifts from James Bamburg [1].

**AAV production**

Using the AVV expression vectors, including pAAV-EGFP, pAAV-cofilin-S3E-EGFP, or pAAV-cofilin-S3A-EGFP, AAV was packed with serotype DJ and concentrated and purified using iodixanol gradient ultracentrifugation by the KIST Virus Facility ([http://virus.kist.re.kr](http://virus.kist.re.kr/)). The titters of each virus were 4.14~6.19 × 10^13^ genome copy/ml (AAV-EGFP), 2.99 × 10^13^ genome copies/ml (AAV-cofilin-S3E-EGFP), and 4.02 × 10^13^ genome copies/ml (AAV-cofilin-S3A-EGFP). AAV expressing EGFP (AAV-EGFP, control), human cofilin-S3E-EGFP (AAV-cofilin-S3E-EGFP), or human cofilin-S3A-EGFP (pAAV-cofilin-S3A-EGFP) were applied to the cultured primary hippocampal neurons or bilaterally injected into the CA1 region of the hippocampi in mouse brains.

**Cell culture and viral infection**

HEK 293 cells were grown in DMEM (Gibco) supplemented with 10% fetal bovine serum (Gibco). Hippocampal neuronal cultures (3 × 10^6^ cells/60 mm) were prepared from E18 Sprague-Dawley rat embryos and maintained for 20 days *in vitro* (DIV) [2]. Cultured hippocampal neurons were infected with 5 μl of AAV-EGFP (6.19 × 10^13^ genome copy/ml) or 5 μl of AAV-cofilin-S3E-EGFP (2.99 × 10^13^ genome copy/ml) at DIV 6, and the cells were collected at DIV 20 for immunoblot analysis.

**Stereotaxic viral injection into the brain**

C57BL/6J mice (4−5-week-old for animal behaviors, 6−8-week-old for electrophysiology) were injected with AAV expressing EGFP, cofilin-S3E-EGFP, or cofilin-S3A-EGFP in the hippocampal CA1 region by stereotaxic surgery. In brief, the mice were anesthetized with isoflurane in an induction chamber, and after confirming proper anesthesia, the animals were fixed in a stereotaxic frame (Stoelting Co.) with ear bars. A 10-μl NanoFil syringe (World Precision Instruments) equipped with a Nanofil 33-gauge blunt needle (World Precision Instruments) was used to inject AAV expressing EGFP, cofilin-S3E-EGFP, or cofilin-S3A-EGFP (0.4 μl per side) into the hippocampal CA1 regions bilaterally at a speed of 0.1 μl/min, based on the anteroposterior and lateral coordinates (anteroposterior, −2.0 mm; mediolateral, ±1.5 mm; dorsoventral, −1.4 mm). Upon completion of the virus injection, the mice were returned to their home cages and allowed to recover. The pairing-induced LTP experiments were performed two weeks after the virus injection, and animal behavior experiments (MWM and delayed nonmatch to place T-maze tasks) were performed 30 days after virus injection. For the pairing-induced LTD experiments, C57BL/6J neonatal mice (P0-1) were injected with AAV expressing EGFP, cofilin-S3E-EGFP, or cofilin-S3A-EGFP using a two-handed procedure, as described previously [3]. In brief, newborn mice were placed on crushed ice for hypothermia-induced anesthesia. After confirming anesthesia, the mice were placed on a custom stage and held by applying moderate pressure with the index finger. A small puncture was made at the needle insertion point using a 30-gauge needle, and AAV expressing EGFP, cofilin-S3E-EGFP, or cofilin-S3A-EGFP (0.2 μl per side) was injected at a speed of 0.1 μl/min using a 10-μl NanoFil syringe (World Precision Instruments) equipped with a Nanofil 33-gauge blunt needle (World Precision Instruments). Following the injection, the mice were returned to their home cages with their mothers and litters. The pairing-induced LTD recordings were performed between 16 and 19 days after the virus injection.

**Preparation of brain homogenates**

Hippocampus, cortex, and forebrain samples obtained from mouse brains were rapidly isolated and homogenized with a Dounce glass tissue grinder homogenizer (Wheaton Industries) in chilled lysis buffer (mM: 50 Tris−HCl, 150 NaCl, 5 EDTA, 1% Triton X-100, protease inhibitor cocktail, 1 PMSF, PhosphoSTOP, pH 7.4) and incubated for 1 h at 4°C. After centrifugation at 1,000*g* for 10 min at 4°C, supernatants were collected, and protein concentrations were measured using Bradford assays (Bio-Rad Protein Assay kit, Bio-Rad Laboratories). The samples were subsequently analyzed by immunoblotting.

**Immunoblot analysis**

Equal quantities of proteins were denatured in 5 × SDS sample buffer (250 mM Tris-HCl, pH 6.8, 10% SDS, 25% β-mercaptoethanol, and 0.05% bromophenol blue) or 4 × Laemmli sample buffer (Bio-Rad, 1610747), separated by SDS-PAGE, transferred onto a PVDF membrane and subjected to immunoblot analysis using anti-GluA1 (Millipore, MAB2263), anti-PSD-95 (Invitrogen, MA1-045), anti-CCNY (Proteintech group, 18042-1-AP; OriGene, TA808174; Atlas antibodies, HPA036290), anti-Synaptophysin (Synaptic systems, 101011), anti-GAPDH (Invitrogen, MA5-15738), anti-β-tubulin (Abcam, ab6046), anti-GFP (Invitrogen, A11122), anti-cofilin (Abcam, ab42824 or Cell Signaling, 5175S), anti-phospho-cofilin (phospho S3, Abcam, ab12866 or Cell Signaling, 3313), anti-LIMK1 (Invitrogen, PA1-31026), and anti-phospho-LIMK1 (Thr508, Invitrogen, PA5-36663) antibodies. Protein bands on immunoblots were visualized using a chemiluminescence method (Millipore) and an imaging documentation system (ImageQuant LAS 4000, GE Healthcare).

**Quantitative real-time PCR and analysis**

The total RNA was extracted from the isolated hippocampus tissues using RNAiso Plus (TaKaRa, Japan) and purified using the RNeasy Plus mini kit (Qiagen). Reverse transcription from the total RNA was carried out using the PrimeScript^TM^ II 1^st^ strand cDNA synthesis kit (TaKaRa, Japan), and the cDNA products were subjected to PCR. The real-time PCR was performed using Power SYBR Green PCR Master Mix (Thermo Fisher Scientific). The reaction mixture contained 500 ng cDNA, 300 nM of each *Ccny* gene-specific primers (forward: 5′- CTGGCAGAAGCAAACAACCT-3′, reverse: 5′-CCTCACAGAGACGAGAGATGG-3′) or *Gapdh* gene-specific primers (forward: 5′-GGGTTCCTATAAATACGGACTGC-3′, reverse: 5′-CCATTTTGTCTACGGGACGA-3′), and 2× Power SYBR Green PCR Master Mix in a total volume of 20 μl. The PCR were performed at 95°C for 10 min, followed by 40 cycles of 95°C for 15 s and 60°C for 1 min, using the StepOnePlus Real-Time PCR System (Applied Biosystems).

**Subcellular fractionation**

Subcellular fractionation was performed as per methods described previously [4, 2]. Briefly, forebrain tissues were isolated and homogenized in buffer A (320 mM sucrose, 20 mM HEPES, 5 mM EDTA, protease inhibitor cocktail, and 1 mM PMSF, pH 7.4) using a glass-teflon homogenizer with 30 strokes. The homogenates (H) were centrifuged for 10 min at 1,000 × *g* to obtain the nuclear fraction (P1), and the remaining supernatant (S1) was further centrifuged for 10 min at 9,200 × *g*. The resulting pellet was washed in buffer A with resuspension, and subsequently centrifuged for 20 min at 10,000 × *g* to produce a crude synaptosomal fraction (P2). The resulting supernatant was further centrifuged at 12,000 × *g* for 30 min to collect the supernatant fraction (S2), which was then further centrifuged using an NVT90 rotor at 165,000 × *g* for 2 h at 4°C to produce the cytosolic supernatant (S3) and the microsomal pellet (P3). The P2 fraction was resuspended in buffer A, and 9 volumes of H_2_O were added to perform lysis via hypo-osmotic shock and 3 strokes using a glass-Teflon homogenizer. The pH was then rapidly adjusted to 7.4 with 4 mM HEPES/5 mM EDTA, and the P2 fraction was further incubated on ice for 30 min. The lysate was then centrifuged at 25,000 × *g* for 20 min at 4°C, which produces the synaptosomal membrane pellet (LP1) and the synaptic vesicle and cytosolic supernatant (LS1). The LS1 fraction was further processed by centrifugation at 165,000 × *g* for 2 h at 4°C using an NVT90 rotor, and the synaptic cytosolic supernatant (LS2) and the synaptic vesicle-enriched pellet (LP2) were obtained. Following the resuspension of LP1, it was loaded on top of a discontinuous sucrose gradient solution (containing 0.8, 1, and 1.2 M sucrose) and further centrifuged at 150,000 × *g* for 2 h at 4°C using an SW41Ti rotor. The cloudy fraction found at the interface of the 1.0 and 1.2 M sucrose gradient was collected carefully and diluted in buffer A. Subsequently, the synaptic plasma membrane fraction (SPM) was obtained by centrifuging the diluted suspension for 30 min at 150,000 × *g* using an SW41Ti rotor. The SPM was resuspended with 0.5% Triton X-100 in buffer A, incubated on ice for 15 min, and divided into soluble (Triton X-100 soluble fraction, T-sol fraction) and insoluble fractions (PSD fraction) by centrifugation at 32,000 × *g* for 20 min. The insoluble PSD fraction was resuspended in buffer A. Protein concentrations were determined using Bradford assays (Bio-Rad Protein Assay kit, Bio-Rad Laboratories), and 5 µg of protein from each fraction was analyzed by immunoblotting.

**Acute hippocampal slice preparation**

WT and *Ccny* KO mice (C57BL/6, male, P17-19 for LFS-induced LTD and pairing-induced LTD, 8-10 weeks old for the rest of the recordings) were anesthetized with halothane, the brain was quickly isolated, and the hippocampi were dissected. Acute hippocampal slices (300 μm for whole-cell, 400 μm for field potential recording) were prepared using a vibratome (Leica, VT1000S) in a chilled cutting buffer containing (in mM) 234 sucrose, 2.5 KCl, 1.25 NaH_2_PO_4_, 24 NaHCO_3_, 11 glucose, 0.5 CaCl_2_, and 10 MgSO_4_ saturated with 95% O_2_ and 5% CO_2_. The hippocampal slices were subsequently recovered at 35°C for 1 h and maintained at room temperature in a recovery artificial cerebrospinal fluid (aCSF) solution containing (in mM) 124 NaCl, 3 KCl, 1.25 NaH_2_PO_4_, 26 NaHCO_3_, 10 glucose, 6.5 MgSO_4_, and 1 CaCl_2_ saturated with 95% O_2_ and 5% CO_2_ until the end of the experiment.

**Electrophysiology**

An acute hippocampal slice was transferred to a recording chamber, and the Schaffer collateral and CA1 neurons were located under a microscope (Olympus, BX51WI) equipped with a CCD camera (Hamamatsu, C3077). The slice was maintained in a recording aCSF solution containing (in mM) 124 NaCl, 3 KCl, 1.25 NaH_2_PO_4_, 26 NaHCO_3_, 10 glucose, 1.3 MgSO_4_, and 2.5 CaCl_2_ saturated with 95% O_2_ and 5% CO_2_, at 30°C throughout the experiments. To measure the paired-pulse ratio (PPR), the Schaffer collateral was stimulated with a 2-contact cluster electrode (FHC, CE2C55), and the stimulation intensity was adjusted using an isolation unit (ISO-Flex, AMPI). A glass pipette filled with aCSF was used to record field excitatory postsynaptic potentials (fEPSPs) in the stratum radiatum layer of CA1. Two consecutive fEPSPs were evoked with various time intervals (25, 50, 100, 200, and 400 ms), and the peak amplitude of the second fEPSP was divided by the first fEPSP to calculate the PPR at a given time interval. To record mEPSCs, 1 μM TTX, 50 μM picrotoxin and 50 μM D-APV were added to the recording aCSF. A whole-cell recording was achieved using glass pipettes of 3-5 MΩ resistance, and the neurons were held at -65 mV under voltage clamp configuration. The internal solution contained (in mM) 125 CsMeSO_3_, 2.8 NaCl, 20 HEPES, 0.4 EGTA, 4 ATP-Mg, 0.5 GTP-Na_2_, 10 phosphocreatine-Na_2_, and 5 QX314 (pH 7.25, 290 mOsm). To record mIPSCs, the recording aCSF was supplemented with 1 μM TTX, 10 μM DNQX, and 50 μM D-APV, and the internal solution contained (in mM) 134 CsCl, 2 MgCl_2_, 10 HEPES, 1 EGTA, 2 ATP-Mg, 0.5 GTP-Na_2_, and 5 phosphocreatine-Na_2_ (pH 7.25, 290 mOsm). The MiniAnalysis software (Synaptosoft Inc.) was used to analyze the recorded mEPSC and mIPSC events with a detection threshold of 8 pA.

To measure NMDAR/AMPAR ratio, 100 μM picrotoxin was added to the recording aCSF. The patch pipettes were filled with an internal solution containing (in mM) 125 CsMeSO_3_, 2.8 NaCl, 20 HEPES, 0.4 EGTA, 4 ATP-Mg, 0.5 GTP-Na_2_, 10 phosphocreatine-Na_2_, and 5 QX314 (pH 7.25, 290 mOsm). The Schaffer collateral was stimulated to evoke synaptic responses, and AMPAR-mediated EPSCs were recorded in CA1 neurons under voltage clamp configuration at a holding potential of -70 mV. After the stable baseline was established, 15 consecutive responses were measured and averaged. Subsequently, the holding potential was increased to +40 mV to record NMDAR-mediated EPSCs. The NMDAR component was determined as the amplitude at 60 ms after stimulation, and the NMDAR/AMPAR ratio was calculated by dividing the averaged value of 15 NMDAR components by the averaged peak amplitude value of AMPAR-mediated EPSCs. To measure the intrinsic neuronal excitability in hippocampal CA1 neurons, 10 μM DNQX, 100 μM picrotoxin, and 50 μM D-APV were added to the recording aCSF. The whole-cell recording was performed under the current clamp configuration, and the internal solution contained (in mM) 130 K-gluconate, 10 KCl, 10 HEPES, 0.2 EGTA, 4 ATP-Mg, 0.5 GTP-Na_2_, and 10 phosphocreatine-Na_2_ (pH 7.25, 290 mOsm). A current step (0 to 330 pA in increments of 30 pA per sweep) was injected to trigger the firing of action potentials. To measure voltage sag responses, a current step ranging from 20 to -150 pA was injected in a stepwise manner, and the input resistance was determined from the linear slope of the current-voltage plots. The sag ratio was calculated by dividing the steady-state response by the peak amplitude.

For the input-output experiments, fEPSPs were evoked by a series of increasing input stimulus delivered at the Schaffer collateral afferents with a 2-contact cluster electrode (FHC, CE2C55). The input-output relationship was determined by plotting the slope of the resulting fEPSP against the presynaptic stimulus intensity, and the ratio of fEPSP slope to the fiber volley amplitude was calculated at the stimulus strength that evokes 40% of the maximal fEPSP amplitude. For field potential recordings of LTP, fEPSPs were evoked by stimulating the Schaffer collateral afferents at 0.033 Hz, and the evoked fEPSPs were recorded in the stratum radiatum layer of CA1 using a glass pipette filled with the standard aCSF. For recording the baseline, the stimulation intensity was adjusted to evoke 40%-60% of the maximal fEPSP amplitude using an isolation unit (ISO-Flex, AMPI). Once a stable baseline is obtained for a minimum of 20 min, LTP was induced by stimulating the Schaffer collateral with high-frequency stimulation (100 Hz trains for 1 s, repeated three times with a 30-s interval). The subsequent changes in the fEPSP slope were monitored for 60 min after LTP induction. For field potential recordings of LTD, single-pulse low-frequency stimulation (900 pulses, 1 Hz) was used to induce LTD. The subsequent changes in the fEPSP slope were monitored for 60 min after LTD induction. For VGCC-LTP experiments, all recordings were performed in the presence of 50 μM D-APV. After collecting a stable baseline of fEPSPs for 20 min at Schaffer collateral-CA1 synapses, four stimulus trains (200 Hz, 0.5 s) were delivered at a rate of 1 train per 5 s. Subsequently, a change in fEPSP slopes was monitored for 60 min. For mGluR-LTD experiments, all recordings were performed in the presence of 50 μM picrotoxin and 5 μM L-689,560. After collecting a stable baseline for 20 min, mGluR-LTD was induced by applying 100 μM DHPG for 10 min. A change in the fEPSP slope was continuously monitored for 60 min after washing out the DHPG.

Pairing-induced LTP and LTD were recorded under the whole-cell patch configuration in acute hippocampal slices prepared from mice injected with AAV-EGFP, AAV-Cofilin-S3E-EGFP, or AAV-Cofilin-S3A-EGFP in CA1 regions or in acute hippocampal slices prepared from WT or *Ccny* KO mice in the presence or absence of Ro25-6981 (Tocris Bioscience, 1594). The slices were continuously perfused with the recording aCSF solution containing (in mM) 124 NaCl, 3 KCl, 1.25 NaH_2_PO_4_, 26 NaHCO_3_, 10 glucose, 1.3 MgSO_4_, 2.5 CaCl_2_, 0.1 picrotoxin, saturated with 95% O_2_ and 5% CO_2_ throughout the experiments. In the case of Ro25-6981-treated recordings, Ro25-6981 (0.5 μM) was applied in the aCSF solution 20 min before the LTP or LTD induction and continuously bath applied till the end of recordings. The recording pipettes were filled with an internal solution containing (in mM) 135 CsMeSO_3_, 8 NaCl, 10 HEPES, 0.3 EGTA, 4 ATP-Mg, 0.3 GTP-Na_2_, 10 phosphocreatine-Na_2_, 5 QX314, and 0.1 spermine (pH 7.25, 290 mOsm). EGFP-positive CA1 neurons were identified under a microscope and whole-cell patched in a voltage-clamp configuration. Subsequently, evoked EPSCs were recorded by stimulating the Schaffer collateral afferents with a 2-contact cluster electrode (FHC, CE2C55) at 0.1 Hz while holding the cell at -65 mV, and a stable baseline was recorded for 5 min. Within 6−7 min of achieving the whole-cell configuration, LTP was induced by stimulating the Schaffer collateral at 2 Hz for 90 s while holding the CA1 neuron at 0 mV. After the pairing stimulation, the holding potential was returned to -65 mV, and the EPSC amplitudes were monitored for at least 40 min. LTD was induced by stimulating the Schaffer collateral at 5 Hz for 3 min while holding the CA1 neurons at -40 mV, and following the pairing, the EPSC amplitudes were monitored for 40 min. All electrophysiology data were collected using a MultiClamp 700B amplifier (Molecular Devices) digitized at 10 kHz with a Digidata 1550 digitizer (Molecular Devices). The Igor Pro (WaveMetrics) and pClamp10 software (Molecular Devices) were used for data acquisition and analysis.

**Open field task**

Mice (male, 8-10 weeks old) were habituated by handling for 10 min a day for three consecutive days. The following day, mice were placed individually in an open-field box (40 × 40 × 40 cm) and allowed to explore freely for 50 min under 50 lux light intensity. The mouse activity was recorded for 50 min by using a top-view video camera and analyzed using the Ethovision XT software (Noldus). The distance moved as well as the time spent in the center zone and the number of entries to the center zone were measured. The center zone was defined as a square within 20 cm in the center of the box.

**Novel object recognition task**

The test was performed in an open-field box (40 × 40 × 40 cm) with the light intensity adjusted to 50 lux. On the day of training, mice (male, 8-10 weeks old) were exposed to two identical objects for 10 min. On the second day 24 h after training, one of the two objects was replaced with a new object, and mice were allowed to explore freely for 10 min. The mouse activity on the second day was recorded by using a video camera, and the exploration time for the old and new objects was analyzed. Exploration behavior was defined as the action of sniffing objects or touching the objects with the nose. The preference index was defined as the ratio of the amount of time spent exploring the new object to the amount of time spent exploring a new or old object. The discrimination index was obtained by dividing the amount of time difference for exploration between the new and the old object by the amount of time spent exploring the new or old object.

**Passive avoidance task**

The apparatus consisted of a black and white chamber (200 × 200 × 200 mm per each) divided by a sliding door. During the training session, each mouse (male, 8−10 weeks old) was placed in the white chamber and allowed to explore for 60 s for habituation. Upon turning on the light in the white chamber, the mouse was allowed to enter the black chamber as the sliding door opened. Once the mouse entered the black chamber, the sliding door was closed, and an electric foot shock (0.35 mA, 1 s) was given through the floor. The mouse was removed from the dark chamber after 10 s. Then, 24 h after the training session, the test session was performed using a protocol identical to that used for the training session but with exclusion of the foot shock procedure. The latency for the mouse to enter the dark chamber was measured at the time point when all four feet of the mouse entered the black room, and the maximum cut-off time for latency was 900 s.

**Morris water maze (MWM) task, reversal leaning, and visible platform test**

A round pool with a diameter of 120 cm was filled with tap water, and a platform (diameter of 10 cm, 0.5−1.0 cm below the water surface) was placed in a quadrant of the pool. White non-toxic paint was added to make the water opaque so that the platform was not visible to the mice. The water temperature was maintained at 21°C-23°C and monitored throughout the experiment. Several visual cues with different shapes were located around the pool so that the mice were able to see them. For the training sessions of the original learning task, mice (male, 8-10 weeks old) were allowed to swim and search for the hidden platform for 90 s. If the mice found the platform within 90 s, they were allowed to stay on the platform for 15 s and were then rescued. If they failed to find the hidden platform within 90 s, they were guided gently to the platform by experimenter’s hand and allowed to stay on the platform for 15 s. Each mouse was trained 4 times a day for 5 consecutive days. The latency to the platform was measured and analyzed. For the probe test on the 6^th^ day, the platform was removed, and the trained mice were allowed to search for the platform for 90 s, 3 times. The latency to the platform zone, travel distance to the platform zone, the frequency of entries to each quadrant, the time spent in each quadrant, and the frequency of passing the platform zone were measured and analyzed. For the reversal learning task performed 24 h after the probe test of the original learning task, the platform was moved to the quadrant opposite the original target quadrant. The mice were subjected to reversal training sessions 4 times a day for three days, and for the reversal probe test on the day 4, the trained mice were allowed to search for the platform for 90 s, 3 times. The latency to the platform for training sessions was measured and analyzed. Latency to the platform zone, travel distance to the platform zone, the time spent in each quadrant, the frequency of entries to each quadrant, and the frequency of passing the platform zone for the probe test were measured and analyzed. For the visible platform test, all visible cues around the platform were removed, and a flag was placed on the platform. The mice were trained for three days, and then the visible probe test was performed on the fourth day. The latency to the platform for training sessions and the latency to the platform zone for the probe test were measured and analyzed. Measurements and analyses of the animal behavior experiments were performed using the EthoVision XT 13 software (Noldus).

**Delayed nonmatch to place T-maze task**

The delayed nonmatch to place T-maze task was performed by using protocols described in previous studies [5, 6] with modifications. Mice (male, 8-9 weeks old) were group-housed and food-deprived by feeding them for 2 h once per day after completing the task. The mice were habituated to a condensed milk reward (1:1 ratio of full-fat sweetened condensed milk to water) in their cage and then in the T-maze apparatus. Mice were handled for two days and then habituated for three consecutive days in the T-maze (start arm: 40 × 10 cm, left and right arms: 35 × 10 cm) with all doors opened in which the condensed milk rewards were placed in both the left and right arms. Daily habituation consisted of two 10-min trials with 6-h intervals. Mice were tested by following acquisition sessions, consisting of four trials per day, where each trial consisted of two runs, including a “forced” run and a “choice” run, with the 5-s interval between forced and choice runs, and 20-min intervals between each trial. At the beginning of the session, both right and left arms were baited with rewards. In the forced run, one randomly selected arm was blocked, and a mouse was placed in the start arm. Because one arm was blocked, the mouse was “forced” to enter the other open arm. After the mouse consumed the reward, it was picked up and placed back into the transfer cage for 5 s (*i.e.*, 5-s delay). In the choice run, both arms were open, thereby allowing the mouse to make a “choice” freely for one arm. Once the tail passed the entrance of one of the arms, the arm was closed by door so that the mouse cannot change its choice. If the mouse chose the opposite arm, where the mouse did not visit during the forced run and the remaining reward still locates, a correct choice was scored. If the mouse chose the same arm visited in the forced run, it was confined to the arm for 10 s without provision of any reward. An inter-trial interval was given for at least 20 min to prevent proactive intervention from the previous trial. The body weights of the mice were maintained at approximately 80% throughout the experiment compared to the initial handling period.

**Intraperitoneal injection of Ro25-6981**

Ro25-6981 was reconstituted at a concentration of 1.2 mg/ml in 0.9% sterile saline and intraperitoneally injected into the mice (6 mg/kg) 1 h before the first trial of each original and reversal training and on probe test days for the MWM task.

**Animal studies**

In all experiments involving animals, the mice were randomly allocated to each group. Animal behavioral experiments and analyses in Figures 2a-o, 4a and b, and 5i-r were performed blind to the investigators.

**RNA sequencing**

The RNA sequencing library was prepared using the TruSeq RNA Sample Prep Kit (Illumina, San Diego, CA, USA, https://www.illumina.com), and sequencing was performed using the Illumina HiSeq 2000 platform to generate 100-bp paired-end reads. The sequenced reads were mapped to the mouse genome (mm10) using STAR (v.2.5.1), and the gene expression levels were quantified using the count module in STAR [7]. The edgeR (v.3.12.1) package was used to quantify gene expression from RNA-seq data [8]. The trimmed mean of M-values (TMM) normalized counts per million (CPM) value of each gene was floored to 1 and log2-transformed for further analysis. The heatmap was constructed using the MeV software [9]. Statistical analyses of RNA-seq data were performed using R (v. 3.3.0) and PYTHON (v. 2.7.6). The mapping statistics for RNA-seq are summarized in Supplementary Table S1.

**Bioinformatic analyses**

Differentially expressed genes (DEGs) were selected based on the following criteria: |fold-change| > 1.5 and *P* < 0.05. The DEGs were subsequently analyzed using the Database for Annotation, Visualization and Integrated Discovery (DAVID) Bioinformatics Resources v6.8 tool ([https://david.ncifcrf.gov](https://david.ncifcrf.gov/)) [10] for the Gene Ontology (GO) analysis and also for the Kyoto Encyclopedia of Genes and Genomes (KEGG) enrichment analysis [11].

**Supplementary References**

1. Garvalov BK, Flynn KC, Neukirchen D, Meyn L, Teusch N, Wu X, et al. Cdc42 regulates cofilin during the establishment of neuronal polarity. J Neurosci. 2007 Nov 28;27(48):13117-29.

2. Hwang H, Hur YN, Sohn H, Seo J, Hong JH, Cho E, et al. Cyclin Y, a novel actin-binding protein, regulates spine plasticity through the cofilin-actin pathway. Prog Neurobiol. 2021 Mar;198:101915.

3. Mathon B, Nassar M, Simonnet J, Le Duigou C, Clemenceau S, Miles R, et al. Increasing the effectiveness of intracerebral injections in adult and neonatal mice: a neurosurgical point of view. Neurosci Bull. 2015 Dec;31(6):685-96.

4. Cho E, Kim D-H, Hur Y-N, Whitcomb DJ, Regan P, Hong J-H, et al. Cyclin Y inhibits plasticity-induced AMPA receptor exocytosis and LTP. Sci Rep. 2015 07/29/online;5.

5. Kellendonk C, Simpson EH, Polan HJ, Malleret G, Vronskaya S, Winiger V, et al. Transient and selective overexpression of dopamine D2 receptors in the striatum causes persistent abnormalities in prefrontal cortex functioning. Neuron. 2006 Feb 16;49(4):603-15.

6. Mills F, Bartlett TE, Dissing-Olesen L, Wisniewska MB, Kuznicki J, Macvicar BA, et al. Cognitive flexibility and long-term depression (LTD) are impaired following beta-catenin stabilization in vivo. Proc Natl Acad Sci U S A. 2014 Jun 10;111(23):8631-6.

7. Dobin A, Davis CA, Schlesinger F, Drenkow J, Zaleski C, Jha S, et al. STAR: ultrafast universal RNA-seq aligner. Bioinformatics. 2013 Jan 1;29(1):15-21.

8. Robinson MD, McCarthy DJ, Smyth GK. edgeR: a Bioconductor package for differential expression analysis of digital gene expression data. Bioinformatics. 2010 Jan 1;26(1):139-40.

9. Howe EA, Sinha R, Schlauch D, Quackenbush J. RNA-Seq analysis in MeV. Bioinformatics. 2011 Nov 15;27(22):3209-10.

10. Huang da W, Sherman BT, Lempicki RA. Systematic and integrative analysis of large gene lists using DAVID bioinformatics resources. Nat Protoc. 2009;4(1):44-57.

11. Kanehisa M, Goto S, Sato Y, Kawashima M, Furumichi M, Tanabe M. Data, information, knowledge and principle: back to metabolism in KEGG. Nucleic Acids Res. 2014 Jan;42(Database issue):D199-205.
