# Supplementary material for: Cyclin Y regulates spatial learning and memory flexibility through distinct control of the actin pathway
Source: Mol Psychiatry. 2022 Nov 25;28(3):1351–64. doi: 10.1038/s41380-022-01877-0 (PMC10005959; doi:10.1038/s41380-022-01877-0)
Supplement: Supplementary file 3 — Supplementary Table S1 [file 41380_2022_1877_MOESM3_ESM.doc]

**Supplementary Table S1. Mapping Statistics for RNA-Seq. Related to Figure 4.**

| **Samples** | **Output reads** | **Uniquely mapped pairs** | **Mapping rate (%)** |
| --- | --- | --- | --- |
| Basal_WT_HC-1 | 42,333,625 | 37,258,175 | 88.01 |
| Basal_WT_HC-2 | 43,290,797 | 37,887,882 | 87.52 |
| Basal_WT_HC-3 | 43,718,531 | 38,139,260 | 87.24 |
| Basal_KO_HC-1 | 45,688,717 | 39,515,696 | 86.49 |
| Basal_KO_HC-2 | 46,976,068 | 40,580,279 | 86.39 |
| Basal_KO_HC-3 | 44,008,739 | 38,615,872 | 87.75 |
| OL_WT_HC-1 | 44,754,979 | 39,473,730 | 88.20 |
| OL_WT_HC-2 | 43,614,895 | 38,332,226 | 87.89 |
| OL_WT_HC-3 | 42,488,301 | 38,217,847 | 89.95 |
| OL_KO_HC-1 | 43,231,208 | 39,126,324 | 90.50 |
| OL_KO_HC-2 | 42,236,197 | 38,012,466 | 90.00 |
| OL_KO_HC-3 | 45,916,562 | 41,634,909 | 90.68 |
| OL-RL_WT_HC-1 | 48,896,764 | 44,103,656 | 90.20 |
| OL-RL_WT_HC-2 | 45,565,891 | 41,460,814 | 90.99 |
| OL-RL_WT_HC-3 | 42,851,837 | 39,027,182 | 91.07 |
| OL-RL_KO_HC-1 | 43,094,733 | 38,731,064 | 89.87 |
| OL-RL_KO_HC-2 | 44,465,564 | 40,093,735 | 90.17 |
| OL-RL_KO_HC-3 | 43,733,216 | 39,400,578 | 90.09 |
